# New Scabimycins A-C Isolated from *Streptomyces acidiscabies* (Lu19992)

**DOI:** 10.3390/molecules26195922

**Published:** 2021-09-29

**Authors:** Constanze Paulus, Josef Zapp, Andriy Luzhetskyy

**Affiliations:** 1 Department of Pharmaceutical Biotechnology, Saarland University, 66123 Saarbrücken, Germany; constanze.paulus@uni-saarland.de; 2 Department of Pharmaceutical Biology, Saarland University, 66123 Saarbrücken, Germany; j.zapp@mx.uni-saarland.de; 3 AMEG Department, Helmholtz Institute for Pharmaceutical Research Saarland, 66123 Saarbrücken, Germany

**Keywords:** *Streptomyces*, peptide natural products, α,β-dehydroamino acid, advanced Marfey’s method

## Abstract

Peptide natural products displaying a wide range of biological activities have become important drug candidates over the years. Microorganisms have been a powerful source of such bioactive peptides, and *Streptomyces* have yielded many novel natural products thus far. In an effort to uncover such new, meaningful compounds, the metabolome of *Streptomyces acidiscabies* was analyzed thoroughly. Three new compounds, scabimycins A–C (**1**–**3**), were discovered, and their chemical structures were elucidated by NMR spectroscopy. The relative and absolute configurations were determined using ROESY NMR experiments and advanced Marfey’s method.

## 1. Introduction

The identification of valuable agents is currently more urgent than ever. The rising drug resistance among disease-causing pathogens is only one reason to drive natural product research forward [1]. Agents for treating cancer, bacterial and viral infections or even neurodegenerative diseases require a source of biologically active compounds, which can lead to new treatment options [2,3,4]. In recent years a subgroup called antimicrobial peptides (AMPs) became a new focus of researchers worldwide due to the often observed enhanced activity against resistant pathogens and the potentially lower risk of resistance development [5]. AMPs are small peptides that consist of 2–15 amino acids (short AMPs) up to 150 amino acids and were found throughout most species (mammalians, insects, amphibians, microorganisms) [6,7,8]. Their differences from conventional antibiotics in terms of pharmacodynamics and their unspecific mode of action, which usually results in slower resistance development, make them a potential alternative treatment option. It was indeed shown over the years that several AMPs or combinations of AMPs/antibiotics can effectively treat multi-drug resistant infections and were thus further developed for clinical trials [9,10,11]. Of course, meanwhile, cases of resistance development are also observed with AMPs, although mechanistically different from antibiotics, which gives hope to have them as back-up when other treatments fail [12]. Through thorough studying of pharmacodynamics or dose-response relationships of AMPs, rapid development of AMP resistant strains can be avoided, which should be of major interest when studying new drug candidates. Yu et al. (2018) and Rodríguez-Rojas (2021) et al. were able to confirm that AMPs have a much steeper pharmacodynamic curve, meaning a higher killing rate in a shorter time frame, and, on the other hand, a rapid increase in concentration while administering AMPs avoids the upcoming of non-inherited resistance caused by tolerant or persistent subpopulations [13,14].

Many AMPs have been isolated from bacteria, e.g., the very first described tyrocidins or gramicidins isolated from *Bacillus brevis* [15] or the well-established nisins [16], and the detection of many more is ongoing. In bacteria, peptides are either ribosomally synthesized and post-translationally modified (RiPPs) or synthesized by large multi-functional enzymes called nonribosomal peptide synthetases (NRPSs) [17,18]. These enzymatic machineries incorporate not only the 20 proteinogenic amino acids, but also many non conventionl amino acids, which causes a huge diversity within peptide structures. Up to 500 different non-proteinogenic amino acid building blocks have been identified so far, including the so-called α,β-dehydroamino acids (dhAAs) [19]. These dhAAs bear a double bond between the α- and the β-carbon of an amino acid and have been often found to be crucial for the activity of a peptide. The physico-chemical properties of dhAAs influence the overall peptide conformation and usually cause the structure to be more rigid, which in turn can have an impact on interaction with targets and thus on the potent activity of certain compounds [20]. Moreover, due to their α,β-double bond, these particular amino acids are more prone to chemical reactions, such as E/Z isomerization, hydrogenation or Michael addition, which is also often the reason why this amino acid contributes significantly to the bioactivity of some compounds [21,22,23].

Herein, we report the isolation and identification of three new peptide compounds that were found in *Streptomyces acidiscabies* (Lu19992) while screening novel actinomycetes strains. Structural elucidation performed using 1D and 2D NMR spectroscopy revealed that these three compounds are linear peptides consisting of acetyl or lactyl starting units and 4–6 amino acids, including proline, alanine, leucine, *allo*-isoleucine and the α,β-dehydroamino acid dehydrobutyrine (dhb). The relative configurations of the dhb moieties were determined using ROESY NMR experiments. Furthermore, the absolute configuration of amino acids was assigned by applying the advanced Marfey’s method. Referring to their origin, those compounds were named scabimycins A–C (**1**–**3**).

## 2. Results

Isolation and Structural Elucidation of Scabimycins A–C

**Figure 1:**
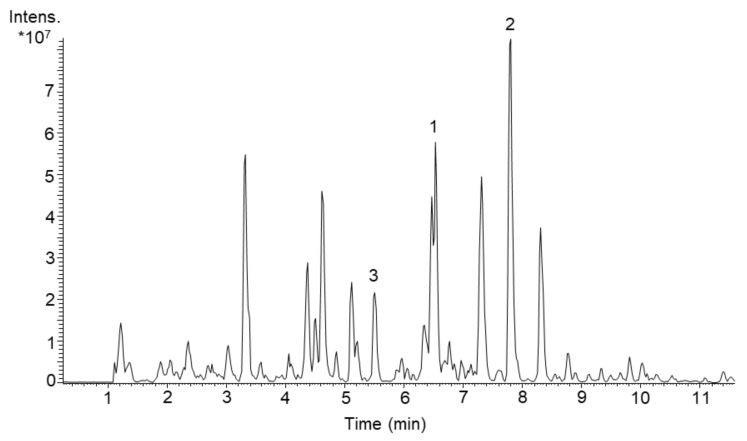
LC-MS chromatogram (RT 1–11 min out of overall 20 min analysis) of butanol extract of S. acidiscabies Lu19992 cultivated in DNPM medium. The peaks representing newly identified compounds are marked: (**1**) Scabimycin A, (**2**) Scabimycin B, (**3**) Scabimycin C.

With the primary goal to discover yet unknown compounds in complex bacterial extracts and subsequent structural identification and confirmation of their novelty, we screened several actinomycetes strains from our library. By comparing exact masses and UV spectra of produced metabolites with the natural product database “Dictionary of Natural Products” (DNP) [24], we evaluated the novelty of each produced compound. In the course of this, the strain *Streptomyces acidiscabies* Lu19992 caught our attention due to the presence of three unknown metabolites (Figure 1). For further characterization of these compounds, we aimed to isolate them and perform structural elucidation. Therefore, *S. acidiscabies* was cultivated in 10 L DNPM medium (production medium consisting of dextrin, bacto soytone, yeast extract and MOPS (4-morpholinepropanesulfonic acid)). The three new compounds **1**–**3** were isolated by extracting the culture broth with butanol, and subsequently purified using flash chromatography, size-exclusion chromatography and as last step semi-preparative HPLC.

**Figure 2:**
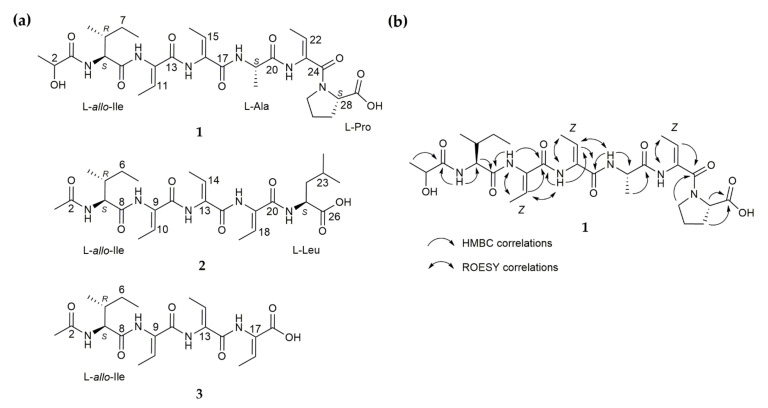
(**a**) Chemical structures of small peptide NPs scabimycins A (**1**), B (**2**) and C (**3**) isolated from *S. acidiscabies* (Lu19992); (**b**) Exemplary for scabimycin A: Key HMBC correlations that were used to assess the peptidic structure and through-space correlations measured by ROESY experiment (Rotational Nuclear Overhauser Effect Spectroscopy) that led to the *Z*-configuration of all dhbs in scabimycins.

Scabimycin A (**1**) was isolated as a light-yellow solid with UV absorbance at 219 nm and a molecular ion peak at *m/z* 621.3229 [M + H]^+^ determined by high-resolution electrospray ionization mass spectrometry (HRESIMS) (Figure S1). The molecular formula C_29_H_44_N_6_O_9_, determined from the exact mass, indicates 11 degrees of unsaturation. The ^1^H NMR spectra acquired in DMSO-*d*_6_ revealed the presence of five protons attached to nitrogen (δ_H_ 9.75, 9.20, 9.06, 7.66, 7.63), eight methine protons (among them, one secondary alcohol and three as part of double bonds; δ_H_ 6.47, 6.17, 5.52, 4.36, 4.33, 4.18, 3.98, 1.83), four methylenes (δ_H_ 3.60–3.40, 1.84–1.72, 2.14–1.77, 1.46–1.06) and seven methyl groups (δ_H_ 1.70, 2 × 1.64, 1.30, 1.19, 0.88, 0.83). Analysis of HSQC and HMBC spectra (no ^13^C spectra were acquired) additionally revealed ten quaternary carbons (seven carbonyls and three double bond carbons). The presence of several NH groups and carbonyls and the typical proton/carbon chemical shifts of amino acid α-CH groups (δ_H/C_ 4.18/58.5, 4.36/48.1, 4.33/55.9) suggest a peptide-like structure consisting of at least three natural amino acids, proline, alanine and isoleucine (Table S1 and Figures S4–S10). The cross peak of methine proton at δ_H_ 6.17 and methyl group at δ_H_ 1.70 in COSY spectrum and their HMBC correlations to the quaternary carbons C-10 (δ_C_ 130.7) and C-13 (δ_C_ 163.5) indicate an α,β-unsaturated amino acid, called dehydrobutyrine (Dhb), of which two additional units were recognized in the NMR spectra. Dehydrobutyrine can be present in the *Z* or *E* configuration; however, it is often found as the *Z* isomer. ROESY correlations indicate the expected *Z*-configuration for all three dhbs (Figure 2 and Figure S6). Further ROESY cross peaks between 3-NH/H-6 and rather weak signals between the 3-NH/H-5 and 3-NH/H-7 groups of isoleucine (Figure S6) lead to the conclusion that the *allo*-version of isoleucine is supposedly present. Finally, a lactyl moiety (Lac) could be recognized by its characteristic chemical shifts (δ_C_ 174.5, δ_H_ 3.98/δ_C_ 66.9, δ_H_ 1.19/δ_C_ 20.6). Careful interpretation of the HMBC correlations led to the peptide sequence Lac-*allo*-Ile-Dhb-Dhb-Ala-Dhb-Pro-OH (Figure 2).

The absolute configuration of scabimycin A was determined using advanced Marfey’s method [25,26]. The compound was hydrolysed and derivatized with D- and L-FDLA and subsequently subjected to LC-HRMS measurements. After careful analysis of retention times and comparison with standards, *allo*-isoleucine, alanine and proline were found to have a L-configuration (Table 1, Figure S19). Under the chosen HPLC conditions of Marfey´s method, the L- and D-forms of isoleucine and *allo*-isoleucine are not clearly distinguishable. Therefore, in addition to the ROESY measurement, we examined the acidic Marfey hydrolysis product by means of ^1^H NMR and compared the spectrum with those of pure L-isoleucine and L-*allo*-isoleucine (Figures S21 and S22). Comparison of the relevant α-protons led without any doubt to the detection of L-*allo* isoleucine.

Scabimycin B (**2**) was isolated as a pale-yellow solid with UV absorbance at 220 nm and a molecular ion peak with *m*/*z* 536.3069 [M + H]^+^ determined by HRESIMS (Figure S2). The molecular formula C_26_H_41_N_5_O_7_, determined from the exact mass, indicates 9 degrees of unsaturation. Again, *allo*-isoleucine could be detected in the 1D and 2D NMR spectra of scabimycin B. However, the Lac moiety was exchanged for an acetyl moiety, which was concluded from the HMBC correlation of H-1 (δ_H_ 1.88/δ_C_ 22.0) to C-2 (δ_C_ 170.8) and the N-HMBC correlation of H-1 to 2-NH (δ_N_ 125.0). It was found that the number and type of amino acids in scabimycin B were slightly different from those found in the amino acid sequence of scabimycin A (Table S1 and Figures S11–S14). Alanine was missing; therefore, isoleucine was followed by three dhbs instead of two; this result is supported by the respective HMBC correlations from 16-NH (dhb-3) to C-16 of the second dhb. Proline was replaced by a terminal leucine, which could be identified by its characteristic shifts. The HMBC correlation from 20-NH to carbonyl C-20 indicates a connection to the third dhb (Figure 2). It was assumed that all the double bonds in dhb were in a *Z* configuration. The absolute configuration was determined using advanced Marfey’s method as described for scabimycin A. Both amino acids, the *allo*-isoleucine and leucine present in scabimycin B, were found to have an L-configuration (Table 1, Figure S20).

Scabimycin C (**3**) was isolated as a yellow, oily compound with a molecular ion peak at *m/z* 423.2227 [M + H]^+^ determined by HRESIMS (Figure S3); however, a fragment at *m*/*z* 322.1755 [M + H]^+^ predominated. Comparison of the NMR data with the previously identified scabimycin B indicated strong similarities. Only the signals of the C-terminal leucine were missing from the spectrum of scabimycin C; this observation is consistent with the identified mass. Thus, **3** is the leucine-truncated version of **2** (Figure 2). The predominant mass of 322 da in the mass spectrum is identical to a fragment lacking a dhb. It is assumed that the relative and absolute stereochemistry is the same as those of scabimycin A and B.

In conclusion, all three new scabimycin natural products contain the allo-version of isoleucine and in total three units of the non-proteinogenic amino acid dhb. However, in scabimycin B and C, allo-Ile and all three dhbs are connected in series whereas in scabimycin A, the series is interrupted through insertion of alanine between the second and the third dhb. Furthermore, in scabimycin A, proline is inserted as the last amino acid in the sequence, whereas scabimycin B ends with leucine. Scabimycin C does not contain any amino acid other than allo-Ile or dhb. Another peculiarity is that scabimycin A has the different starting unit lactyl instead of the acetyl unit found in scabimycin derivatives B and C. The differences in the amino acid sequence suggest a flexible extender unit choice of employed enzymes during biosynthesis. To explain the differences in the amount and order of amino acids and also the choice of the different starting units, it would be necessary to take a closer look at the underlying biosynthetic machinery of these compounds.

Scabimycin A–C were tested on antimicrobial and antifungal activity. No activity was observed against Gram-positive Bacillus subtilis or Gram-negative Pseudomonas putida and E. coli strains. We also tested the ability of the newly purified compounds to inhibit 3CL protease (SARS-CoV-2). However, none of the scabimycins inhibited the protease activity.

## 3. Discussion

It is more important than ever to find new natural products to expand the libraries for the development of useful medicines. While screening new actinobacteria strains, a streptomyces strain named *Streptomyces acidiscabies* Lu19992 attracted our attention due to extensive production of three putatively unknown metabolites. These compounds were isolated and characterized via NMR spectroscopy resulting in three novel short linear peptides that harbor the rather unusual amino acid dhb. This amino acid arises from dehydration of threonine, which occurs either through action of a dehydratase enzyme or via the elimination of phosphorylated residues [27]. Dhbs are often found in ribosomally synthesized and post-translationally modified peptides (RiPPs), e.g., in thiopeptides such as cyclothiazomycin [28] or in depsipeptides such as tumescenamides [29], but also in peptides synthesized by NRPS, e.g., in albopeptin or in the recently discovered malpinins [30,31]. The genome of *S. acidiscabies* harbors several NRPS and RiPPs biosynthetic gene clusters, which became apparent after genome analysis using the antiSMASH online tool [32]. Detailed analysis of these clusters did not give a clear hint if any of them could be responsible for the production of scabimycin compounds. It would be necessary to perform gene deletion or heterologous expression experiments to identify the correct biosynthetic gene cluster and also to unveil the reasons for the choice of different starting units (lactyl/acetyl) during biosynthesis and the different amino acid sequence of scabimycin A contrary to B and C. Scabimycins were not found to possess antibacterial activity against representative Gram-positive or Gram-negative bacteria tested in our laboratory. To certainly exclude antibacterial activity of these compounds, a wider range of pathogenic bacteria needs to be tested. For example the recently discovered tripeptide albopeptin, which contain also the amino acid dhb, was found to be active against the clinical isolate of vancomycin-resistant *Enterococcus faecium* K60–39 [30].

Additionally, new compounds should be tested more thoroughly, considering that different conditions while testing antimicrobial activity have a major impact on susceptibility of tested bacteria. As it was nicely summarized in a review by Mercer et al. (2020), if certain AMPs do not perform well under “standard” laboratory test conditions developed for antibiotics, it does not mean that there is not potential activity. It has been proven that AMPs show a lack or attenuation of antimicrobial activity when tested using conditions for conventional antibiotic assays. Various factors can influence antimicrobial activity of AMPs under in vitro testing, e.g., pH, temperature, buffer, nutrient concentrations, charge effects, and growth phase to name a few. Thus, to test the real activity of new drug candidates, standard procedures for antimicrobial susceptibility testing need to be adjusted [33]. For example, it was confirmed that pH regulation within bacterial population can vary significantly due to the presence of persister or viable but non-culturable cells and thus the response to antibiotic treatments [34,35]. Considering new insights into behavior of bacterial subcultures will have an impact on how activity testing can be improved.

The vast application possibilities of short peptides were just recently extensively reviewed by Apostolopoulos et al. (2021) [36]. Referring to this overview, scabimycins could be used alternatively in many different areas. Short peptides are used, for example, in gene delivery systems as alternative targeting moiety that lowers the risk of immunogenicity or as cell penetrating peptides [37,38]. It is also worth noting that (short) peptides have a high impact in the development of vaccines used for the treatment of various infections and cancer [39,40]. Furthermore, peptides are currently also developed to treat neurodegenerative diseases [41]. Considering this wide range of pharmaceutical applications, it becomes clear that peptides will play an important role in the future development of valuable therapeutics. Thus, to ensure that we have a basis to work on, it is important to continue identifying more interesting peptides with various length and types of incorporated amino acids. Bacteria are prolific sources of important peptide molecules, and the continuous screening of interesting new strains can hopefully help to broaden the supply of useful peptides. Herein, we showed that screening of a new streptomyces strain led to the discovery of three new short peptides with an unusual amino acid sequence that can be added to the pool of basic structures for the development of future medicines.

## 4. Materials and Methods

### 4.1. Cultivation, Metabolite Extraction and Dereplication

The S. acidiscabies strain was precultivated in a 100 mL flask filled with 10 mL of TSB (tryptic soy broth 30 g/L) at 28 °C and 180 rpm on a rotary shaker for 24 h. For the main culture, 50 mL of DNPM medium (dextrin 40 g/L, bacto soytone 7.5 g/L, yeast extract 5 g/L and MOPS (4-morpholinepropanesulfonic acid) 21 g/L, pH 6.8) was inoculated in a 500 mL flask with 1 mL of preculture and cultivated for 5 days at 28 °C and 180 rpm on a rotary shaker. After cultivation, the biomass and culture liquid were extracted separately with a mixture of acetone/methanol (1:1) and butanol, respectively. The solvent was evaporated until the residue was dry, and then the residue was dissolved in 300 µL MeOH. High-resolution LC-MS data were collected on a Dionex Ultimate 3000 UHPLC system (Thermo Fisher Scientific, Waltham, MA, USA) with stationary phase Acquity UPLC BEH C18, 100 mm × 2.1 mm, 1.7 µm column (Waters Corporation, Milford, MA, USA) using a linear gradient from 5 % [B] (acetonitrile + 0.1 % formic acid) against [A] (ddH_2_O + 0.1 % formic acid) to 95 % [B] and coupled to a PDA detector operating at 200–600 nm. The coupled LTQ Orbitrap mass spectrometer (Thermo Fisher Scientific, Waltham, MA, USA) was operated at *m/z* 200–2000. Data were analyzed with Thermo Xcalibur Version 3.0.63 software. Dereplication was performed by comparing the exact masses with the Dictionary of Natural Products database version 10.0 (CRC Press, Boca Raton, FL, USA).

### 4.2. Isolation, Purification and Structure Elucidation

For isolation and purification of the targeted compounds, *S. acidiscabies* was cultivated in 10 L of DNPM (100 × 500 mL flasks with 100 mL medium) as described above. After cultivation, the culture liquid was separated from the biomass and extracted twice with butanol. The solvent was removed under reduced pressure, and the residue was dissolved in methanol and centrifuged. The pellet was discarded, and the supernatant was dried, resulting in 16 g of raw material. The crude extract was dissolved in 20 mL methanol and fractionated by flash chromatography (Isolera™ One, Biotage, Uppsala, Sweden) using a Chromabond^®^ Flash RS 330 C18 ec 360 g column (Macherey-Nagel, Düren, Germany). Two runs (10 mL of extract for each) were performed using Milli-Q^®^ (MQ) (Merck Millipore, Burlington, MA, USA) -H_2_O [A] and methanol [B] as eluents. A linear gradient from 30–80% [B] over 5 column volumes (CV) was applied. Fractions were tested on LC-MS, a Dionex Ultimate 3000 UPLC system using Acquity BEH C18, 50 × 2.1 mm, 1.7 µm d_p_ column (Waters Corporation, Milford, MA, USA) and mobile phase: ddH_2_O + 0.1% formic acid [A]/acetonitrile + 0.1% formic acid [B], 5–95% [B] over 9 min, at flow rate 0.6 mL/min, coupled to amaZON SL speed mass spectrometer (Bruker, Billerica, MA, USA) with ESI source and mass range *m/z* 200–2000. Fractions containing the targeted compounds were pooled and further purified with size exclusion chromatography using Sephadex^®^ LH 20 (Sigma Aldrich, Germany) as the stationary phase (50 cm column, filled with a 300 mL volume of Sephadex in methanol) and methanol as the eluent. Fractions were collected every 15 min with a speed of 1–2 drops per second. Every third fraction was analyzed by LC-MS (Bruker amaZon speed, see above). The fractions containing scabimycins were further purified by semipreparative high-performance liquid chromatography (HPLC) using the following equipment: Agilent 1100 and 1260 Series HPLC (Agilent Technologies, Santa Clara, CA, USA) equipped with a Nucleodur C18 HTEC column (250 × 4.6 mm, 5 µm, Macherey-Nagel, Düren, Germany) and a DAD detector operating at 200–600 nm. A linear gradient used solvent [A] MQ-H_2_O + 0.1% formic acid against solvent [B] acetonitrile + 0.1 % formic acid starting from 35% [B] and increasing to 45% [B] over 20 min with a flow rate of 4.5 mL/min at 45 °C.

NMR spectra were acquired in deuterated dimethyl sulfoxide (DMSO-*d*_6_) at 298 K on a Bruker Avance III 700 or 500 MHz spectrometer, both equipped with a 5 mm TXI cryoprobe. The NMR shifts were relative to the position of the residual solvent signal of DMSO-*d*_6_ at δ 2.50 ^1^H, or to the solvent itself at δ 39.5 (DMSO-*d*_6_) for ^13^C measurements. NMR data were analysed using Topspin, version 3.5 pl7 (Bruker, Billerica, MA, USA) and Spectrus Processor 2018.2.3 (ACD/Labs, Canada).

Scabimycin A (**1**). Lac-l-*allo*-Ile-Dhb-Dhb-l-Ala-Dhb-l-Pro-OH. Yellow oil; 5.0 mg; ${\left[\alpha \right]}_{D}^{20}$ − 34.4 (c 0.9 mg/mL, MeOH); UV (MeOH) λ_max_ 219 nm; δ_H_ (700 MHz, DMSO-*d*_6_): 9.75 (s, NH-9), 9.20 (s, NH-20), 9.06 (s, NH-13), 7.66 (d, NH-3), 7.63 (d, NH-17), 6.47 (q, H-15), 6.17 (q, H-11), 5.52 (q, H-22), 4.36 (t, H-18), 4.33 (t, H-4), 4.18 (t, H-28), 3.98 (q, H-2), 3.60–3.40 (m, 2xH-25), 2.14–1.77 (m, 2xH-27), 1.84–1.72 (m, 2xH-26), 1.83 (m, H-5), 1.70 (d, 3xH-12), 2 × 1.64 (d, 3xH-16/3xH-23), 1.46–1.06 (m, 2xH-7), 1.30 (m, 3xH-19), 0.89 (t, 3xH-6), 0.84 (t, H-8); δ_C_ (700 MHz, DMSO-*d*_6_): 174.5 (C-3), 173.2 (C-29), 2 × 170.9 (C-9, C-20), 165.3 (C-24), 163.6 (C-17), 163.5 (C-13), 131.4 (C-21), 130.7 (C-10), 129.9 (C-14), 129.1 (C-15), 124.8 (C-11), 120.2 (C-22), 66.9 (C-2), 58.5 (C-28), 55.9 (C-4), 48.5 (C-25), 48.1 (C-18), 36.8 (C-5), 28.8 (C-27), 24.8 (C-26), 23.9 (C-7), 20.6 (C-1), 17.0 (C-19), 15.2 (C-6), 2 × 12.4 (C-16, C-12), 11.8 (C-23), 10.9 (C-8); HRESIMS *m*/*z* 621.3229 [M + H]^+^ (calc. for C_29_H_44_N_6_O_9_ ) (meas. 621.3209 [M + H]^+^, calc. 621.3247 [M + H]^+^).

Scabimycin B (**2**). Ac-l-*allo*-Ile-Dhb-Dhb-Dhb-l-Leu-OH. Yellow oil; 1.8 mg; ${\left[\alpha \right]}_{D}^{20}$ − 44.2 (c 2.65 mg/mL, MeOH); UV (MeOH) λ_max_ 220 nm; δ_H_ (700 MHz, DMSO-*d*_6_): 9.63 (s, NH-8), 9.00 (s, NH-12), 8.67 (s, NH-16), 8.42 (s, NH-2), 7.42 (d, NH-20), 6.49 (q, H-18), 6.43 (q, H-10), 6.32 (q, H-14), 4.18 (m, H-21), 4.06 (m, H-3), 1.88 (s, 3xH-1), 1.77 (m, 3xH-4), 1.69 (d, 3xH-11), 1.68 (d, 3xH-15), 1.62 (m, 3xH-23), 1.61 (d, 3xH-19), 1.60–1.48 (m, 2xH-22); 1.55–1.19 (m, 2xH-6), 0.92 (d, H-5), 0.86 (t, H-7), 0.84 (d, 3xH-24), 0.81 (d, 3xH-25); δ_C_ (700 MHz, DMSO-*d*_6_): 173.6 (C-26), 171.3 (C-8), 170.8 (C-2), 163.7 (C-12), 163.0 (C-20), 162.6 (C-16), 130.3 (C-13), 2 × 129.8 (C-17/C-9), 129.4 (C-18), 128.3 (C-10), 127.5 (C-14), 58.2 (C-3), 50.5 (C-21), 39.8 (C-22); 34.8 (C-4), 24.5 (C-6), 23.7 (C-23), 22.6 (C-24), 22.0 (C-1), 20.9 (C-25), 15.1 (C-5), 3 × 12.6 (C-11/C-15/C-19), 10.7 (C-7); HRESIMS *m*/*z* 536.3069 [M + H]^+^ (calc. for C_26_H_41_N_5_O_7_) (meas. 536.3068 [M + H]^+^, calc. 536.3084 [M + H]^+^).

Scabimycin C (**3**). Ac-l-*allo*-Ile-Dhb-Dhb-Dhb-OH. Yellow oil; 2.0 mg; ${\left[\alpha \right]}_{D}^{20}$ − 60.7 (c 1.45 mg/mL, MeOH); UV (MeOH) 220 nm; δ_H_ (700 MHz, DMSO-*d*_6_): 9.61 (s, 8-NH), 8.78 (s, 12-NH), 8.44 (s, 16-NH), 8.23 (d, 2-NH), 6.49 (m, H-14), 6.48 (m, H-18), 6.36 (q, H-10), 4.06 (t, H-3), 1.86 (s, 3xH-1), 1.73 (m, H-4), 1.69 (d, 3xH-11), 2 × 1.63 (d, 3xH-15/3xH-19), 1.52–1.18 (m, 2xH-6), 0.91 (d, H-5), 0.85 (t, 3xH-7); δ_C_ (700 MHz, DMSO-*d*_6_): 171.7 (C-8), 170.4 (C-2), 163.2 (C-12), 2 × 162.4 (C-20/C-16), 130.7 (C-9), 2 × 130.1 (C-18/C-14), 2 × 129.2 (C-13/C-17), 127.6 (C-10), 58.4 (C-3), 35.5 (C-4), 25.1 (C-6), 22.0 (C-1), 15.4 (C-5), 2 × 13.8 (C-15/C-19), 13.1 (C-11), 11.1 (C-7); HRESIMS *m*/*z* 423.2227 [M + H]^+^ (calc. for C_26_H_41_N_5_O_7_) (meas. 423.2227 [M + H]^+^, calc. 423.2243 [M + H]^+^).

For determination of absolute configuration using advanced Marfey’s method, samples **1** (200 µg) and **2** (250 µg) were treated with 100 µL of a 6 M HCl solution and heated at 110 °C for 45 min. Subsequently, the solvent was removed completely under a nitrogen stream at the same temperature within 20 min. The residues were dissolved in 110 µL H_2_O, and 50 µL was transferred into two new tubes for each sample. To each tube, 20 µL of 1 M NaHCO_3_ was added to reach pH 9. For derivatization, the samples were treated with 20 µL of 1% Marfey’s reagent (D-FDLA and L-FDLA, respectively) in acetone. Similarly, 20 µL of 1 M NaHCO_3_ and 20 µL of a 1% solution of L-FDLA were added to 50 µL of an aqueous solution (50 mmol) of each present amino acid (D/L-proline, D/L-alanine, D/L-leucine, L-isoleucine). All samples were then incubated for 1.5 h at 40 °C and 700 rpm. To stop the reaction, 10 µL of 2 M HCl solution was added. Afterwards, 300 µL acetonitrile was added, and the samples were subjected to LC-HRMS measurements. Samples were run on an Acquity BEH C18, 100 × 2.1 mm, 1.7 µm d_p_ column (Waters Corporation, Milford, MA, USA) using eluent [A] ddH2O + 0.1% formic acid and [B] acetonitrile + 0.1% formic acid. The following gradient was applied: 5–10% [B] (0–2 min), 10–25% [B] (2–15 min), 25–50% [B] (15–22 min), 50–95% [B] (22–24 min), 95 -95% [B] (24–26 min), and 95–5% [B] (26–27 min). The column oven temperature was set to 45 °C, and a mass range from *m*/*z* 100–1000 was acquired.

## Figures and Tables

**Table 1:** Retention times of amino acids in scabimycins A and B after derivatization with D-FDLA and L-FDLA.

	Scabimycin A			Scabimycin B	
	*allo*-Ile	Ala	Pro	*allo*-Ile	Leu
D-FDLA	23.2 min	20.1 min	19.8 min	23.2 min	23.3 min
L-FDLA	20.7 min	18.4 min	18.6 min	20.7 min	20.9 min

## Data Availability

Not applicable.

## Supplementary Materials

The following are available online: Table S1: NMR spectroscopic data of Scabimycins A-C (**1**–**3**) in DMSO-*d*_6_, Figure S1: MS spectra of scabimycin A, showing the exact mass *m*/*z* 621.3228 [M + H]^+^, Figure S2: MS spectra of scabimycin B, showing the exact mass *m*/*z* 536.3067 [M + H]^+^, Figure S3: MS spectra of scabimycin C, showing the exact mass without dehydrobutyrine (as explained in the main text, this mass predominates) *m*/*z* 322.1753 [M + H]^+^, Figure S4: ^1^H NMR spectrum of scabimycin A (DMSO-*d*_6_, 700 MHz), Figure S5: ^1^H-^1^H-Cosy spectrum of scabimycin A (DMSO-*d*_6_, 700 MHz), Figure S6: ROESY spectrum of scabimycin A (DMSO-*d*_6_, 700 MHz), Figure S7: HSQC spectrum of scabimycin A (DMSO-*d*_6_, 700 MHz), Figure S8: HMBC spectrum of scabimycin A (DMSO-*d*_6_, 700 MHz), Figure S9: ^15^N-HSQC spectrum of scabimycin A (DMSO-*d*_6_, 700 MHz), Figure S10: ^15^N-HMBC spectrum of scabimycin A (DMSO-*d*_6_, 700 MHz), Figure S11: ^1^H NMR spectrum of scabimycin B (DMSO-*d*_6_, 700 MHz), Figure S12: ^1^H-^1^H-Cosy spectrum of scabimycin B (DMSO-*d*_6_, 700 MHz), Figure S13: HSQC spectrum of scabimycin B (DMSO-*d*_6_, 700 MHz), Figure S14: HMBC spectrum of scabimycin B (DMSO-*d*_6_, 700 MHz), Figure S15: ^1^H NMR spectrum of scabimycin C (DMSO-*d*_6_, 700 MHz), Figure S16: ^1^H-^1^H-Cosy spectrum of scabimycin C (DMSO-*d*_6_, 700 MHz), Figure S17: HSQC spectrum of scabimycin B (DMSO-*d*_6_, 700 MHz), Figure S18: HMBC spectrum of scabimycin C (DMSO-*d*_6_, 700 MHz), Figure S19: Section of LC-HRMS chromatogram (retention time 17 to 28 min) of hydrolysed scabimycin A derivatized with D-FDLA and L-FDLA showing single amino acids D/L-alanine (1/2), D/L-proline (3/4) and isoleucine (5) and leucine (1/3). To compare the amino acids present in our probe, pure D/L-amino acids derivatized with L-FDLA (D/L-alanine (1*/2*), D/L-proline (3*/4*) and L-isoleucine (5*)) are shown below. Comparison with the standard amino acids and the later elution of the D-FDLA-derivatized amino acids lead us to the assumption that in all cases, the amino acids possess an L-configuration. It needs to be mentioned that from this test, it is not possible to decide whether L-allo-isoleucine or L-isolelucine is present. The intense peak at RT 19.5 coincides with the mass of the underivatized FDLA, Figure S20: Section of LC-HRMS chromatogram (retention time 17 to 28 min) of hydrolysed scabimycin B derivatized with D-FDLA and L-FDLA showing single amino acids isoleucine (2) and leucine (1/3). To compare the amino acids present in our probe, pure D/L-amino acids derivatized with L-FDLA (D-leucine (1*), L-leucine (3*) and L-isoleucine (2*)) are shown below. Comparison with the standard amino acids and the later elution of the D-FDLA-derivatized amino acids led us to the assumption that in both cases, the amino acids have an L-configuration. It needs to be mentioned that from this test, it is not possible to decide whether L-allo-isoleucine or L-isolelucine is present. The intense peak at RT 19.5 coincides with the mass of the underivatized FLDA, Figure S21: Determination of allo-isoleucine in scabimycin A. Scabimycin A, allo-L-Ile and L-Ile were treated with HCl at 110 °C. ^1^H-NMR were acquired in DMSO-*d*_6_ and compared. The chemical shifts of the α-protons of scabimycin A and allo-L-Ile coincide, while the α-proton of L-Ile is shifted upfield, Figure S22: HSQC spectrum of scabimycin A after treatment with HCl; this spectrum supports the findings for allo-isoleucine. The ^13^C chemical shift of the α-carbon was found to be approximately 55 ppm, as expected for isoleucine.
