# Supplementary material for: New Scabimycins A-C Isolated from Streptomyces acidiscabies (Lu19992)
Source: Molecules. 2021 Sep 29;26(19):5922. doi: 10.3390/molecules26195922 (PMC8513078; doi:10.3390/molecules26195922)
Supplement: Supplementary file 1 [file molecules-26-05922-s001.zip › molecules-1338502-supplementary.pdf]

Supporting Information

Table S1. NMR spectroscopic data of scabimycins A-C (**1**-**3**) in DMSO-d_6

Scabimycin A (1)

position	δ_C[a]	δ_H[b]	Cosy	HMBC	N-HMBC	N-HSQC	NOESY
1	20.6, CH_3	1.19, d (6.8)	2	2, 3	-	-	-
2	66.9, CH	3.98, q (6.8)	1	1, 3	-	-	-
3	174.5, C	-	-	-	-	-	-
				***allo*-Ile**			
3-NH	-	7.66, d (7.6)	4	3, 4, 9	-	111.3	9-NH
4	55.9, CH	4.33, t (7.6)	5, 3-NH	5, 6, 7	3-NH	-	9-NH
5	36.8, CH	1.83, m	4, 6	4, 6, 7, 8	-	-	-
6	15.2, CH_3	0.89, t (6.6)	5	4, 5, 7			-
7	23.9, CH_2	1.46, m 1.06, m	8	4, 5, 6, 8	-	-	-
8	10.9, CH_3	0.84, t (7.4)	7	5, 7	-	-	-
9	170.9, C	-	-	-	-	-	-
				**Dhb**			
9-NH	-	9.75, s	-	9, 13	-	124.8	3-NH, 4, 12
10	130.7, C	-	-	-	-	-	-
11	124.8, CH	6.17, q (6.9)	12	10, 12, 13	9-NH	-	13-NH
12	12.4, CH_3	1.70, d (6.9)	11	10, 11	-	-	9-NH
13	163.5, C	-	-	-	-	-	-

Scabimycin B (2)

position	δ_C[a]	δ_H[b]	Cosy	HMBC	N-HMBC	N-HSQC
1	22.0, CH_3	1.88, s	-	2	2-NH	-
2	170.8, C	-	-	-	-	-
			***allo*-Ile**			
2-NH	-	8.42, s (br)	3	-	-	125.0
3	58.2, CH	4.06, m	2-NH, 4	4, 5, 6, 8	2-NH	-
4	34.8, CH	1.77, m	3, 5	-	-	-
5	15.1 CH_3	0.92, d (6.9)	4	3, 4, 6	-	-
6	24.5 CH_2	1.55, m 1.19, m	7	3, 4, 7	-	-
7	10.7, CH_3	0.86, t*	6	4, 6	-	-
8	171.3, C	-	-	-	-	-
			**Dhb**			
8-NH	-	9.63, s	11	8, 12	-	123.6
9	129.8, C	-	-	-	-	-
10	128.3, CH	6.43, q (7.0)	11	9, 11, 12	8-NH	-
11	12.6, CH_3	1.69, d (7.0)	10	9	-	-
12	163.7, C	-	-	-	-	-

Scabimycin C (3)

position	δ_C[a]	δ_H[b]	Cosy	HMBC
1	22.0, CH_3	1.86, s	-	2
2	170.4, C	-	-	-
		***allo*-Ile**		
2-NH	-	8.23, d (6.8)	3	2, 3, 4
3	58.4, CH	4.06, t (6.8)	2-NH	4, 5, 6, 8
4	35.5, CH	1.73, m	3, 5	-
5	15.4 CH_3	0.91, d (6.6)	4	3, 4, 6
6	25.1 CH_2	1.52, m 1.18, m	7	4, 5, 7
7	11.1 CH_3	0.85, t (7.4)	6	4, 6
8	171.7, C	-	-	-
		**Dhb**		
8-NH	-	9.61, s	11	8, 10, 12
9	130.7, C	-	-	-
10	127.6, CH	6.36, q (7.2)	11	11, 12
11	13.1, CH_3	1.69, d (6.9)	8-NH, 10	9, 10
12	163.2, C	-	-	-

**Dhb**								**Dhb**							**Dhb**				
13-NH	-	9.06, s	-	13, 15	-	115.7	11, 16, 17-NH	12-NH	-	9.00, s	15	12, 16	-	116.5	12-NH	-	8.78, s	15	14, 16
14	129.9, C	-	-	-	-	-	-	13	130.3, C	-	-	-	-	-	13	129.2, C	-	-	-
15	129.1, CH	6.47, q (6.9)	16	16, 17	13-NH	-	17-NH	14	127.5, CH	6.32, q (7.0)	15	13, 16	12-NH	-	14	130.1, CH	6.49, m	15	15, 16
16	12.4, CH_3	1.64, d (6.9)	15	14, 15	-	-	-	15	12.6, CH_3	1.68, d (7.0)	14	13, 14	-	-	15	13.8, CH_3	1.63, d (6.7)	12-NH, 14	13, 14
17	163.6, C	-	-	-	-	-	-	16	162.6, C	-	-	-	-	-	16	162.4, C	-	-	-
**Ala**								**Dhb**							**Dhb**				
17-NH	-	7.63, d (7.0)	18	17, 18, 19	-	116.4	13-NH, 19, 20-NH	16-NH	-	8.67, s	19	16, 18	-	112.4	16-NH	-	8.44, s	19	20
18	48.1, CH	4.36, t (7.0)	17-NH, 19	17, 19	17-NH	-	20-NH	17	129.8, C	-	-	-	-	-	17	129.2, C	-	-	-
19	17.0, CH_3	1.30, (7.0)	18	18, 20	17-NH	-	-	18	129.4, CH	6.49, q (7.0)	19	17, 19, 20	16-NH	-	18	130.1, CH	6.48, m	19	19, 20
20	170.9, C	-	-	-	-	-	-	19	12.6, CH_3	1.61, d (7.0)	18	18, 20	-	-	19	13.8, CH_3	1.63, d (6.7)	16-NH, 18	17, 18
								20	163.0, C	-	-	-	-	-	20	162.4, C	-	-	-
**Dhb**								**Leu**											
20-NH	-	9.20, s	23	20, 24	-	121.7	17-NH, 18, 19, 23	20-NH	-	7.42, d (7.8)	21	20, 21	-	115.1					
21	131.4, C	-	-	-	-	-	-	21	50.5, CH	4.18, m	20-NH, 22	20, 22, 23, 26	-	-					
22	120.2, CH	5.52, q (6.9)	23	21, 23, 24	20-NH	-	25	22	39.8, CH_2	1.60, m<br>1.48, m	21, 23	21, 23, 26	20-NH	-					
23	11.8, CH_3	1.64, (6.9)	22	21, 22	-	-	-	23	23.7, CH	1.62, m	22, 24, 25	22, 26	-	-					
24	165.3, C	-	-	-	-	-	-	24/25	20.9, CH_3<br>22.6, CH_3	0.81, d (6.5)<br>0.84, d (6.5)	23	22, 23	-	-					
**Pro**																			

25	48.5, CH_2	3.60, m<br>3.40, m	25	26, 27, 28	-	-	-
26	24.8, CH_2	1.84, m<br>1.72 m	25, 27	28	-	-	-
27	28.8, CH_2	2.14, m<br>1.77, m	26, 28	25, 26	-	-	-
28	58.5, CH	4.18, t (7.4)	27	26, 27, 29	-	-	-
29	173.2, C	-	-	-	-	-	-

[a]followed by multiplicity, ^{13}C chemical shifts were all taken from HSQC and HMBC spectra
[b]followed by multiplicity and coupling constant *J* in Hz

MS spectra of scabimycins A-C

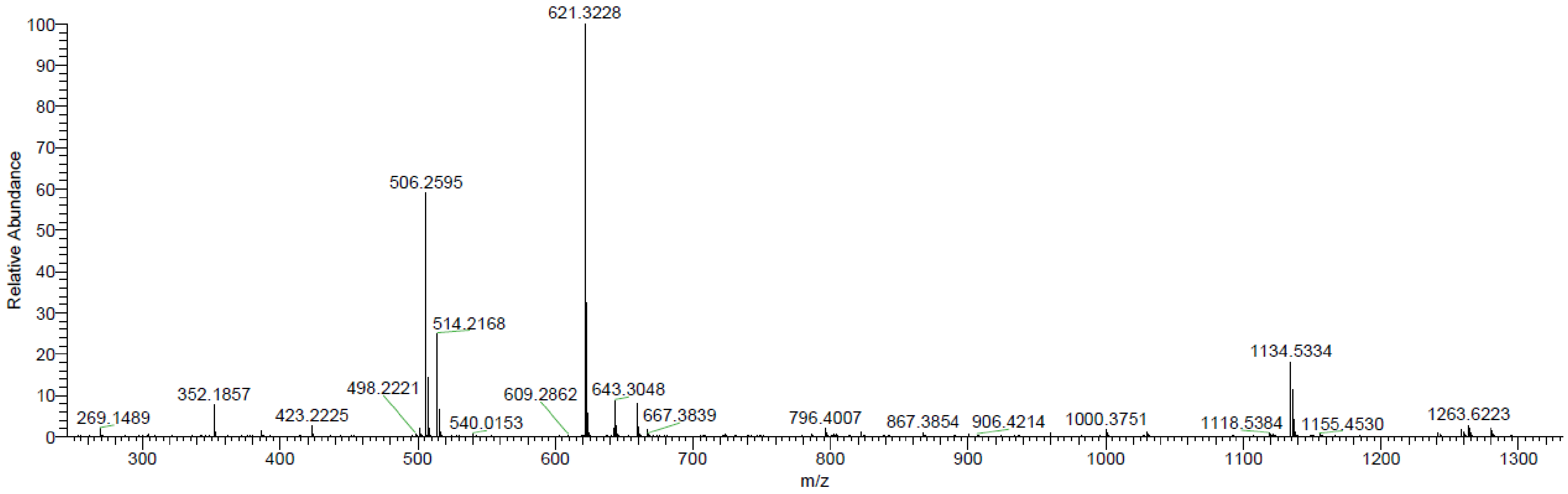

Figure S1. MS spectra of scabimycin A, showing the exact mass m/z 621.3228 [M+H]$^+$.

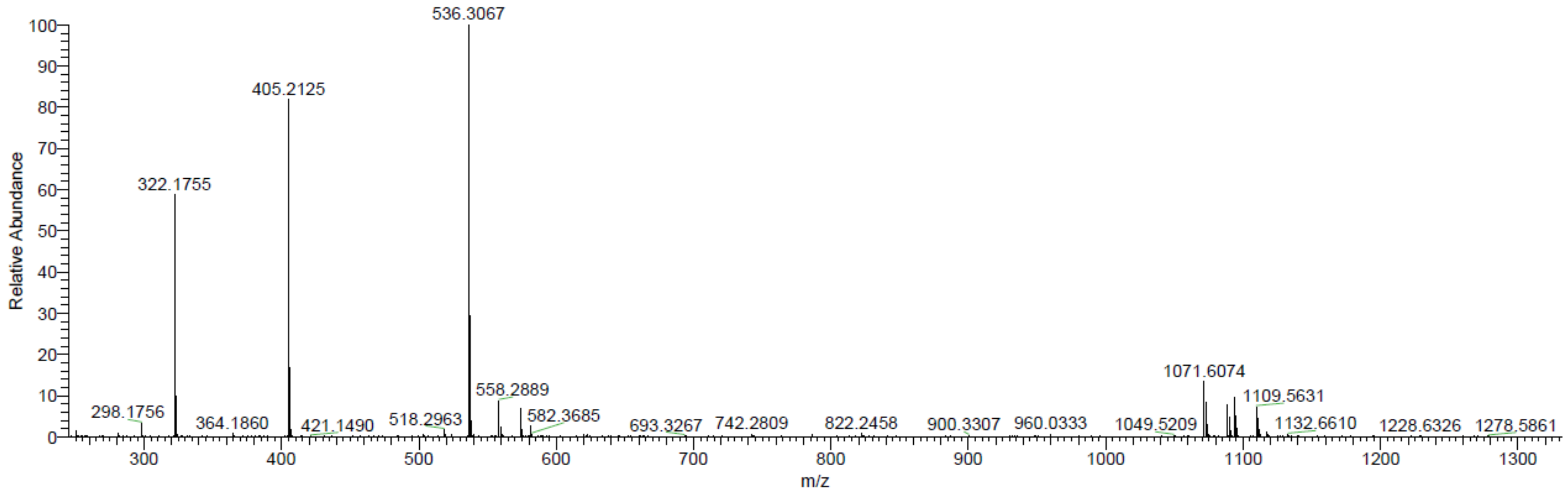

Figure S2. MS spectra of scabimycin B, showing the exact mass m/z 536.3067 [M+H]+.

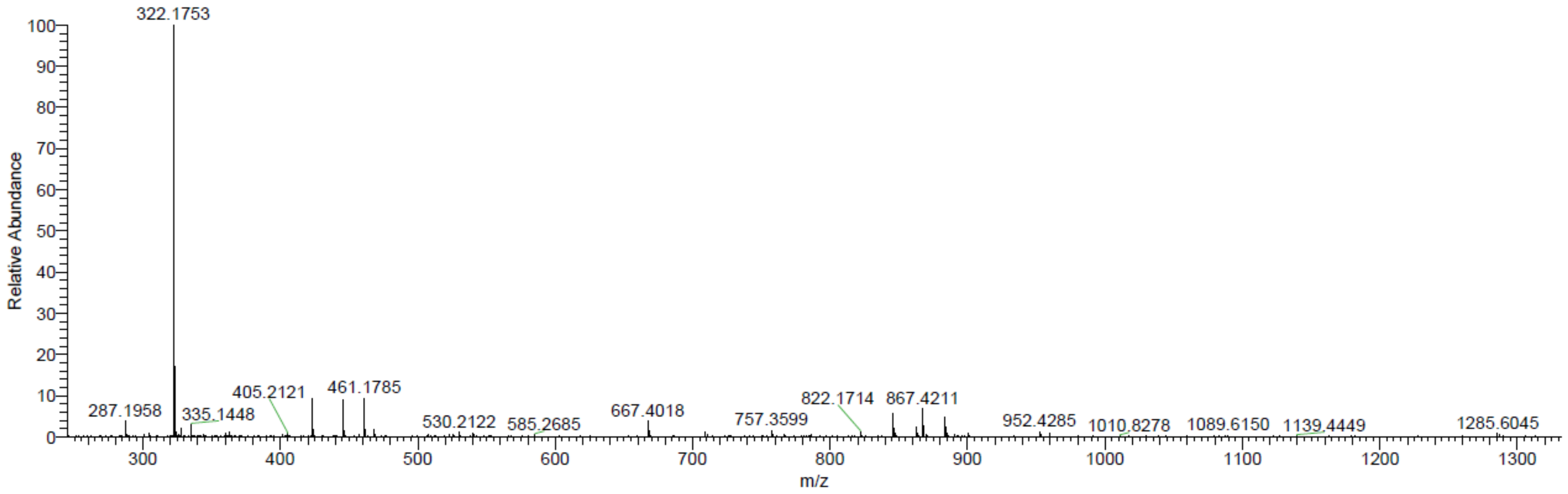

Figure S3. MS spectra of scabimycin C, showing the exact mass without dehydrobutyrine (as explained in the main text, this mass predominates) m/z 322.1753 [M+H]+.

1D/2D NMR spectra of Scabimycins A-C

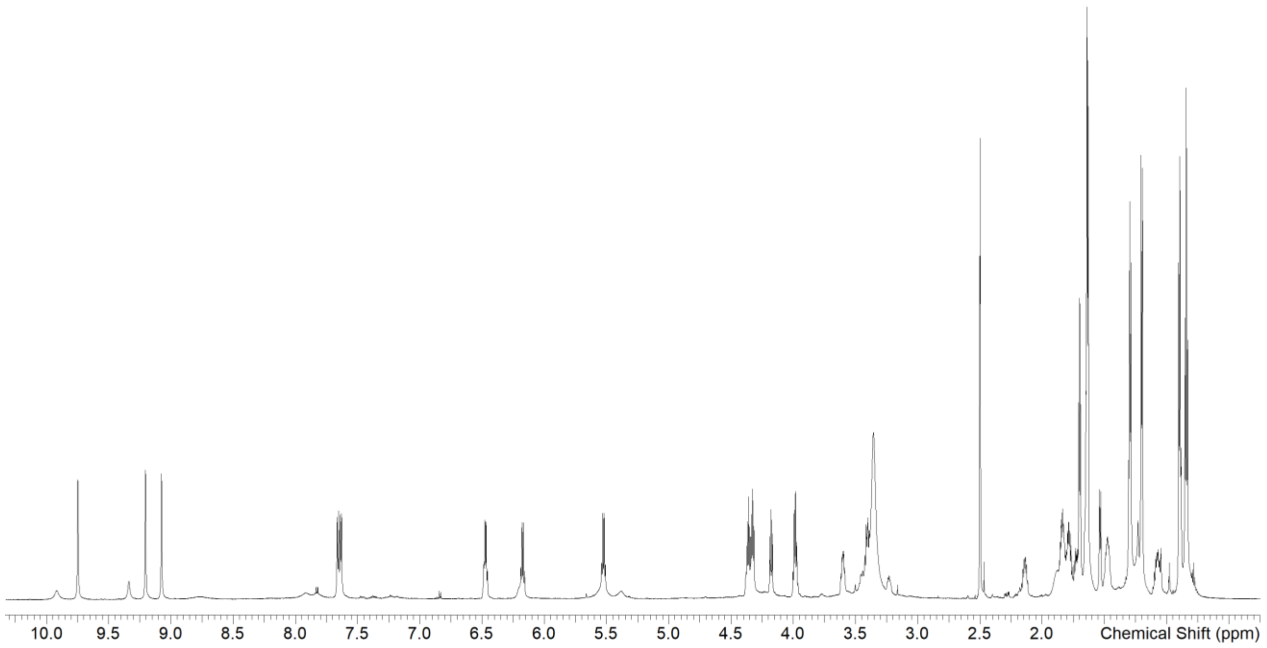

Figure S4. ^{1}H NMR spectrum of scabimycin A (DMSO-d_6, 700 MHz).

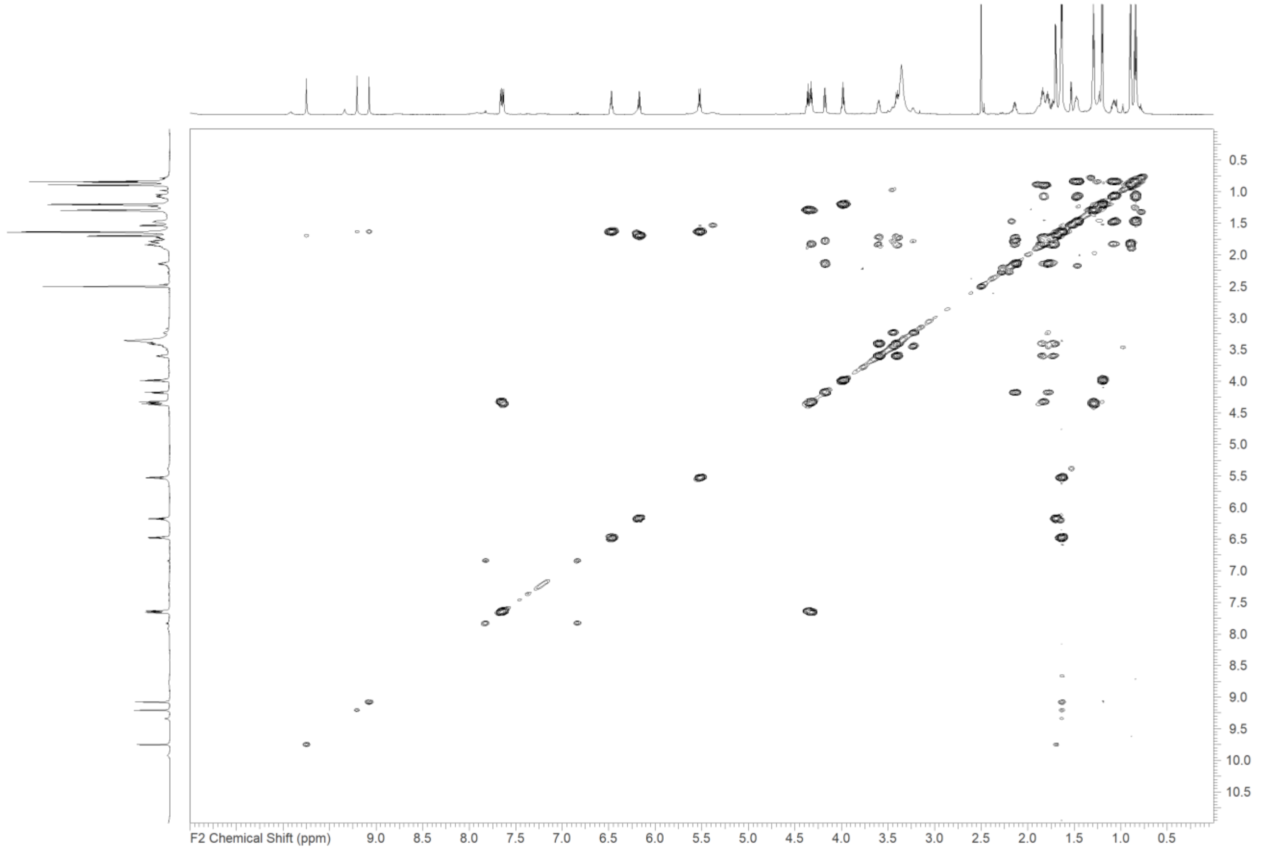

Figure S5. ^{1}H-^{1}H-Cosy spectrum of scabimycin A (DMSO-d_6, 700 MHz).

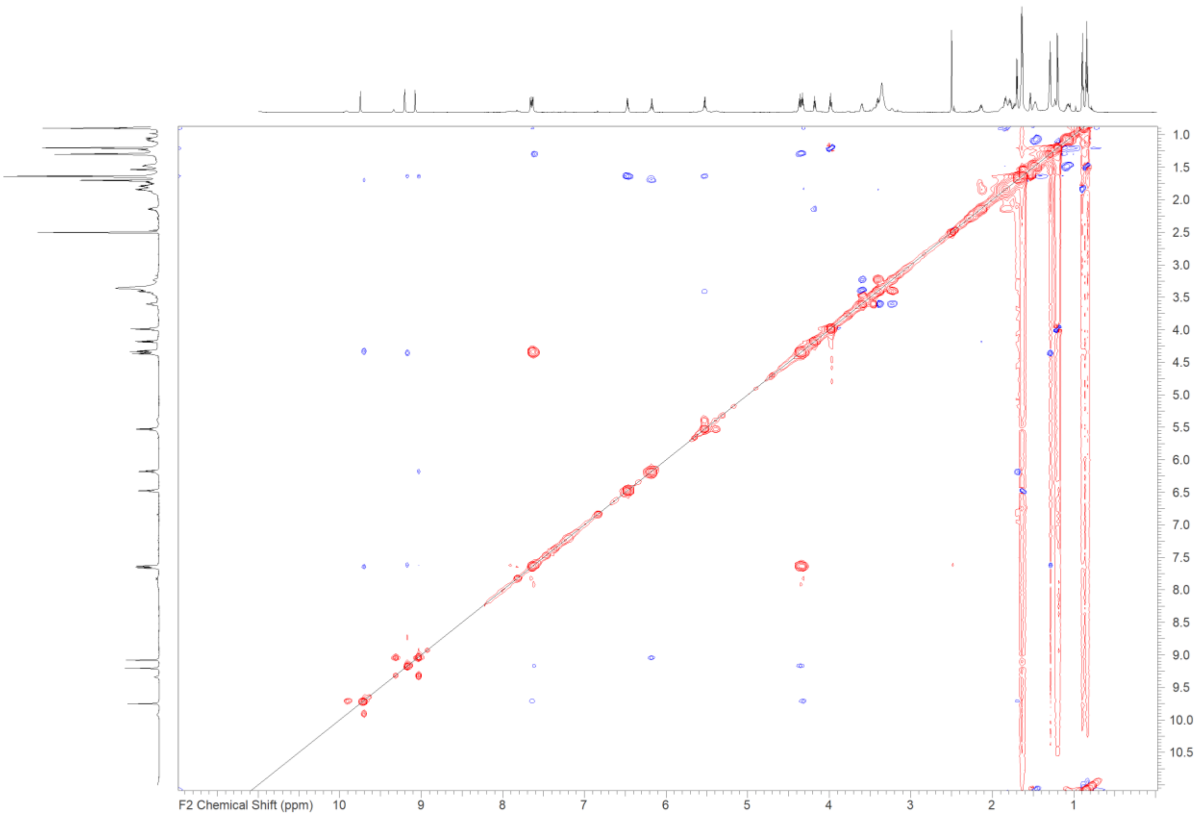

Figure S6. ROESY spectrum of scabimycin A (DMSO-d6, 700 MHz).

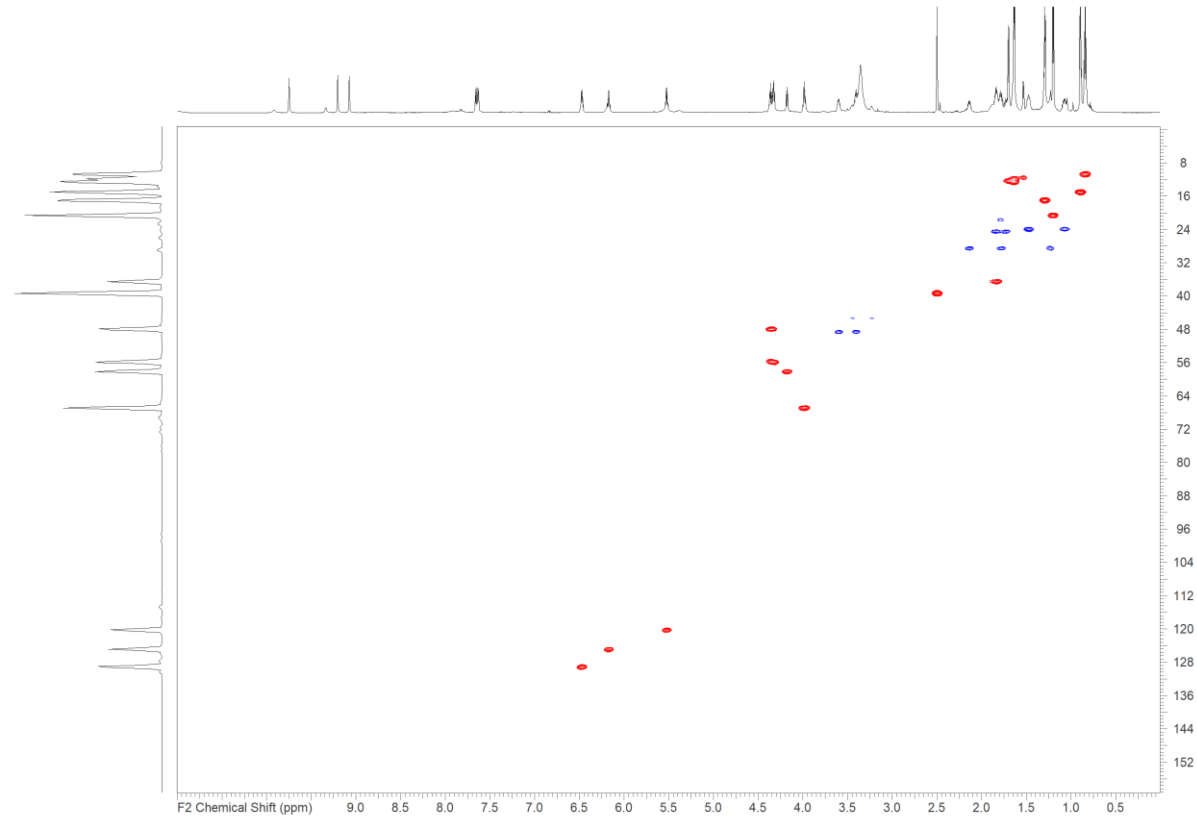

Figure S7. HSQC spectrum of scabimycin A (DMSO-d6, 700 MHz).

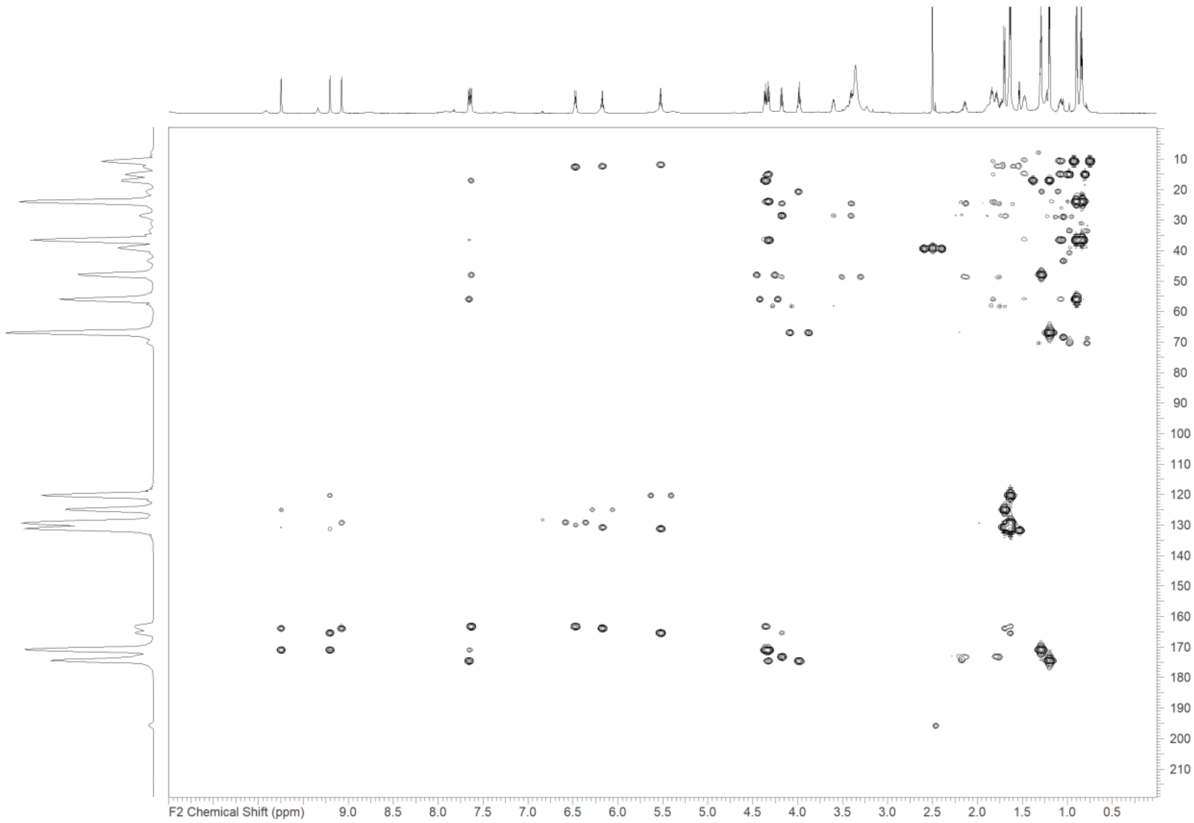

Figure 8. HMBC spectrum of scabimycin A (DMSO-d₆, 700 MHz).

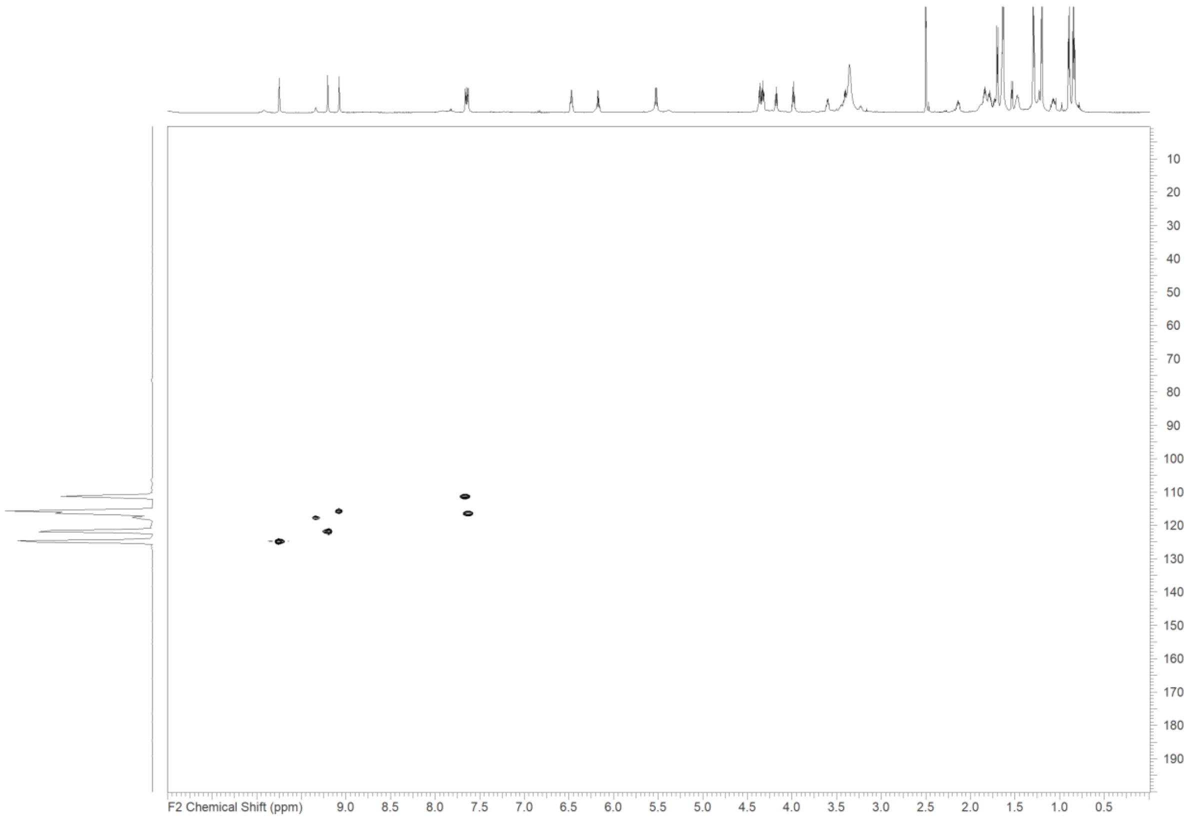

Figure S9. ^{15}N-HSQC spectrum of scabimycin A (DMSO-d$_6$, 700 MHz).

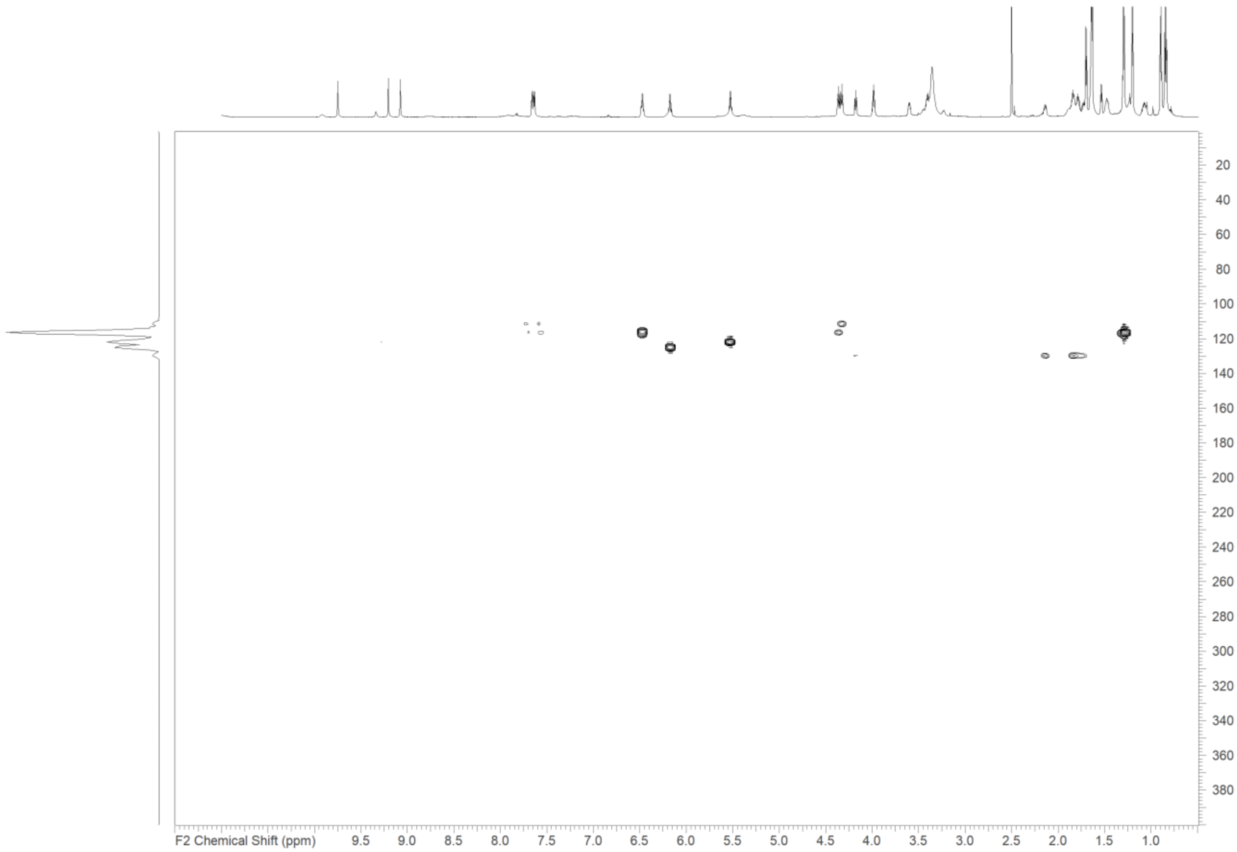

Figure S10. ^{15}N-HMBC spectrum of scabimycin A (DMSO-d$_6$, 700 MHz).

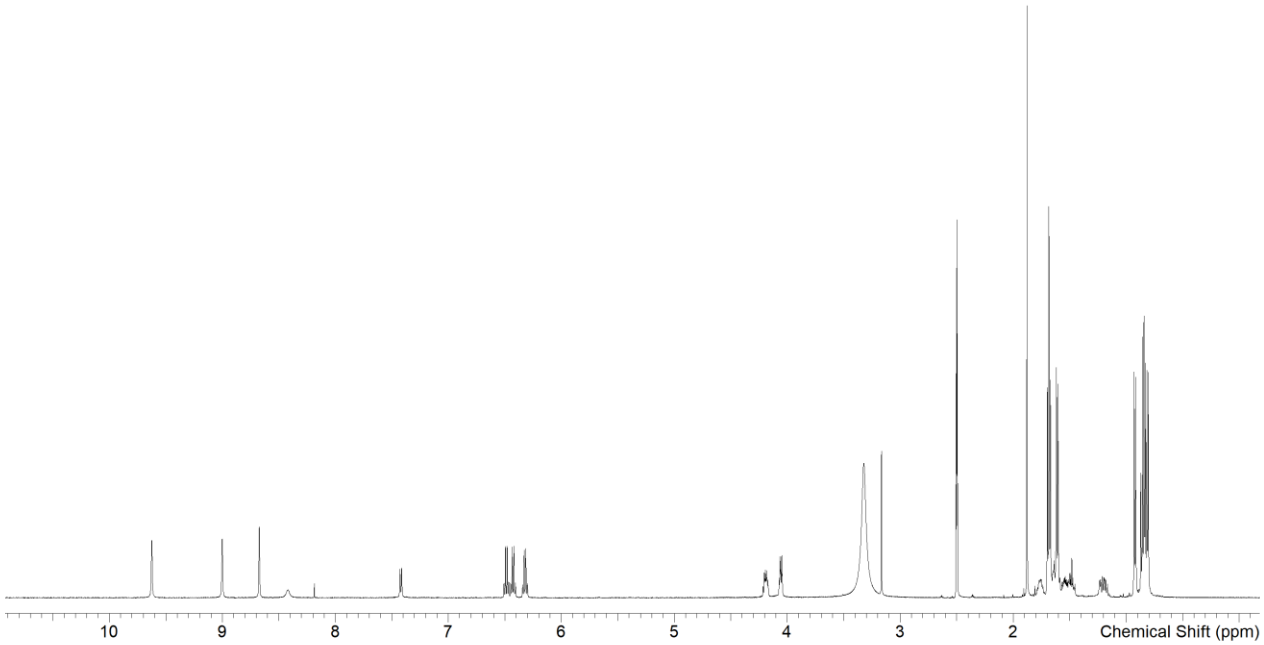

Figure S11. ^{1}H NMR spectrum of scabimycin B (DMSO-d$_6$, 700 MHz).

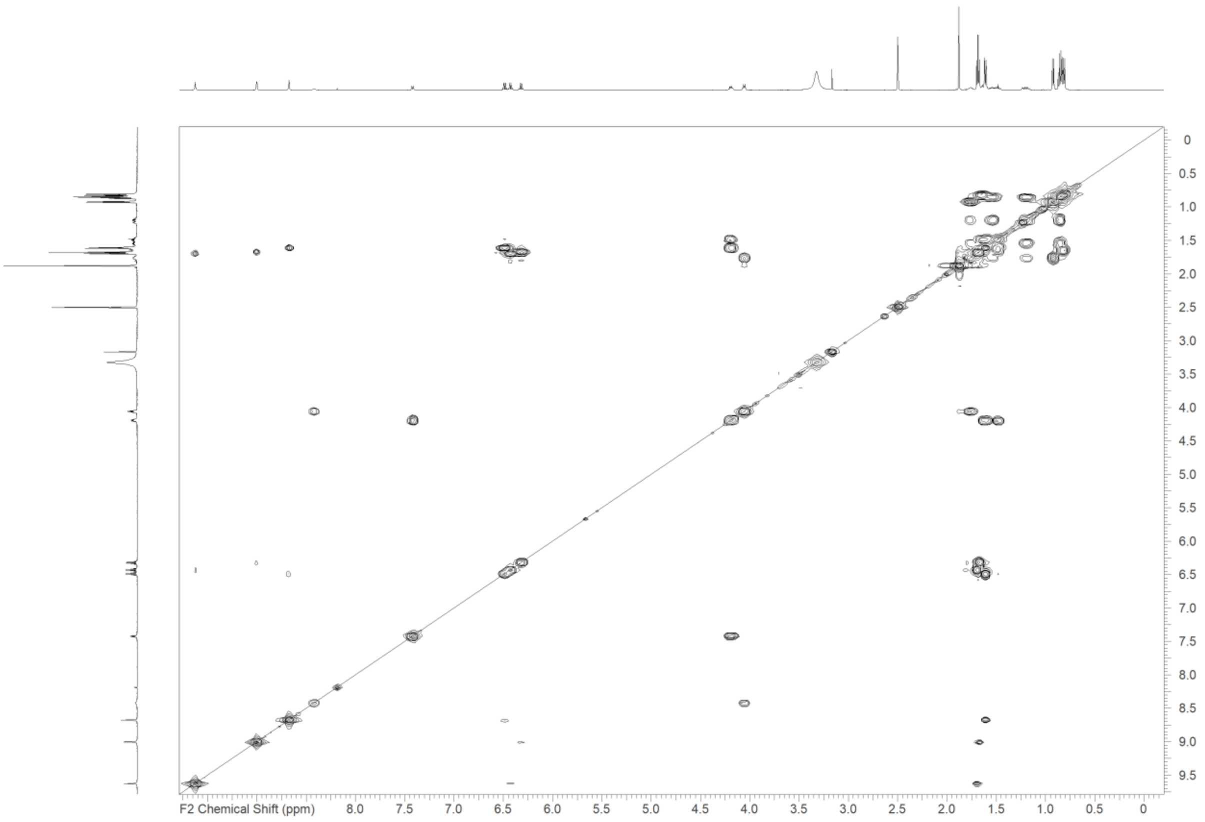

Figure S12. ^{1}H-^{1}H-Cosy spectrum of scabimycin B (DMSO-d_6, 700 MHz).

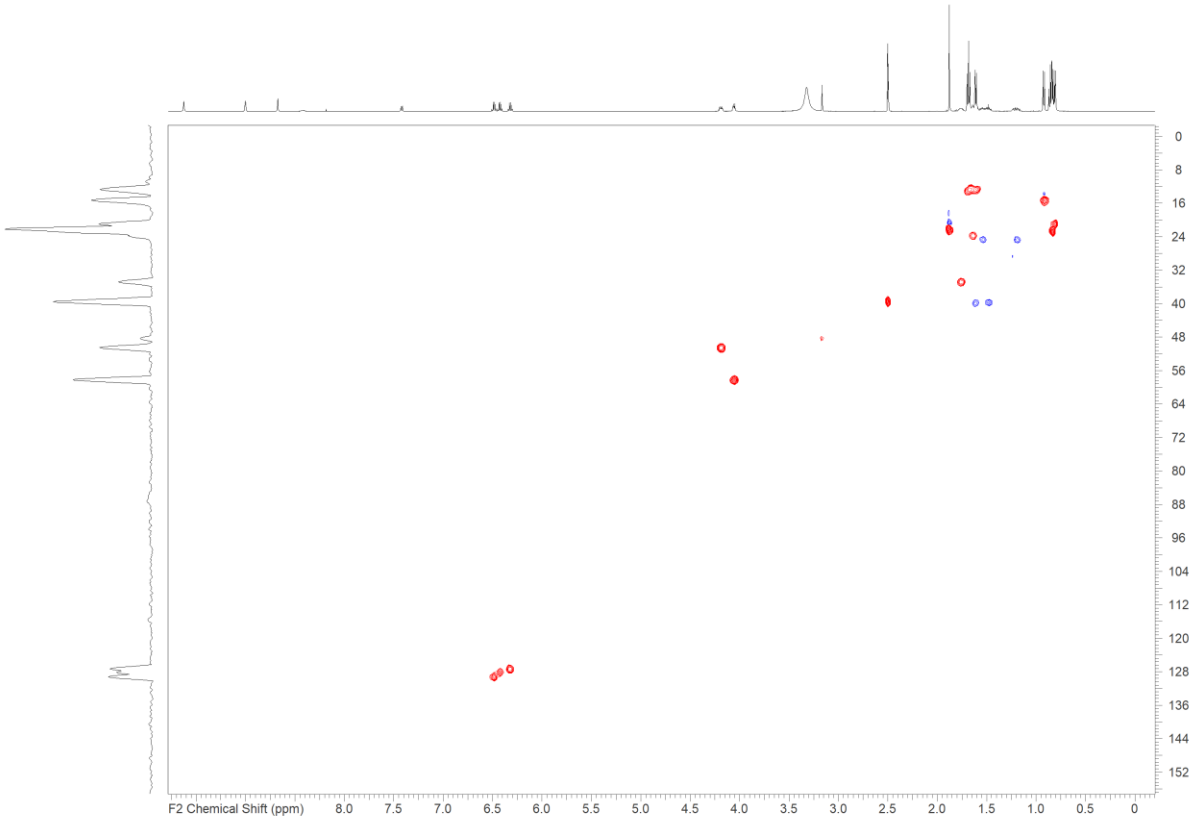

Figure S13. HSQC spectrum of scabimycin B (DMSO-d_6, 700 MHz).

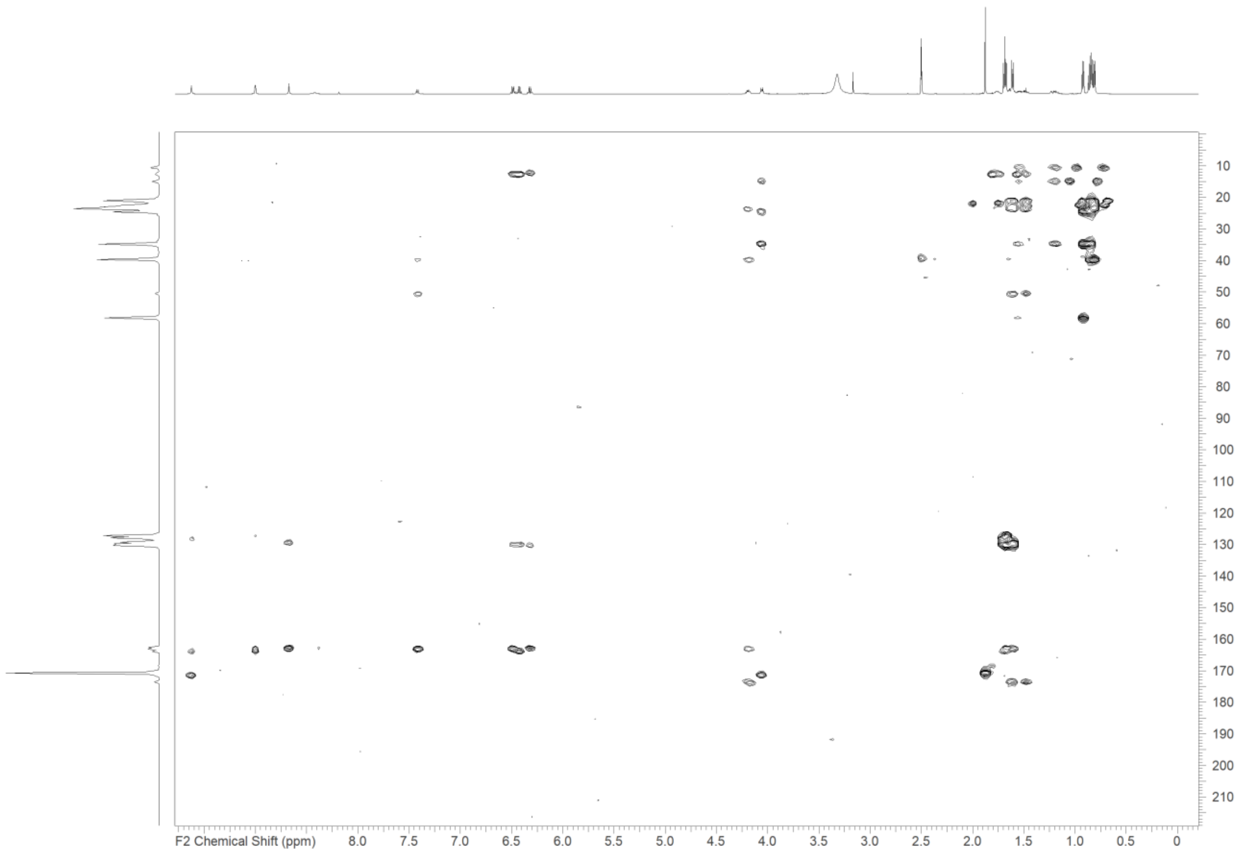

Figure S14. HMBC spectrum of scabimycin B (DMSO-d_6, 700 MHz).

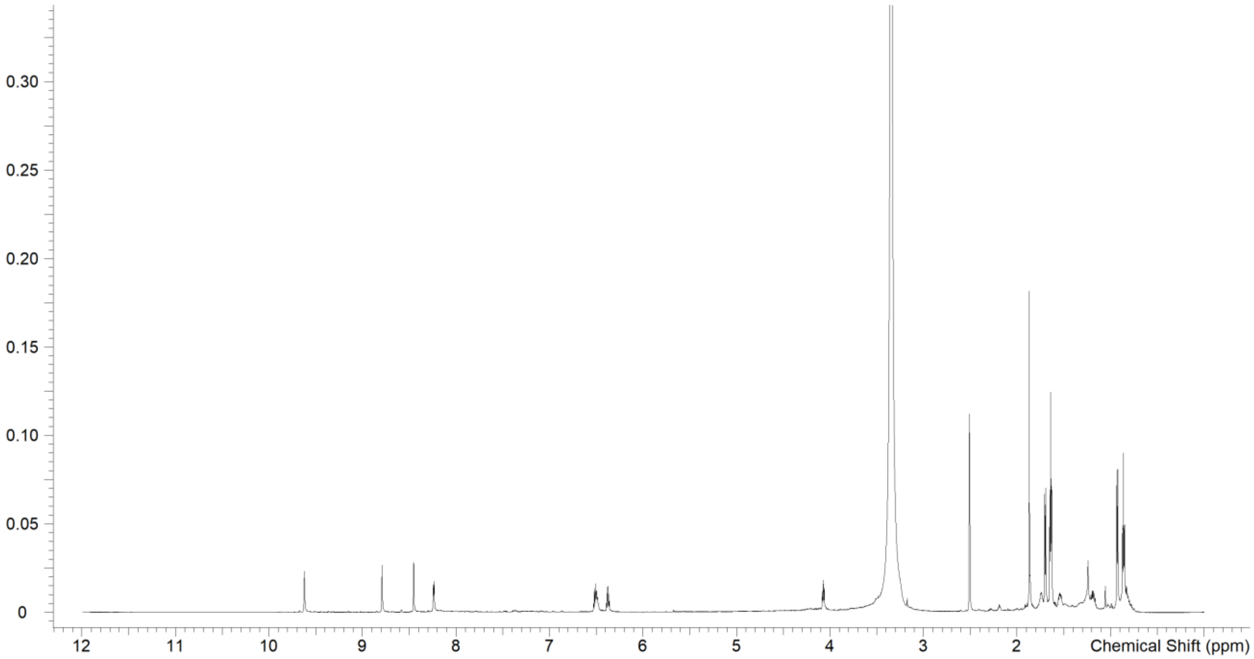

Figure S15. ^{1}H NMR spectrum of scabimycin C (DMSO-d_6, 700 MHz).

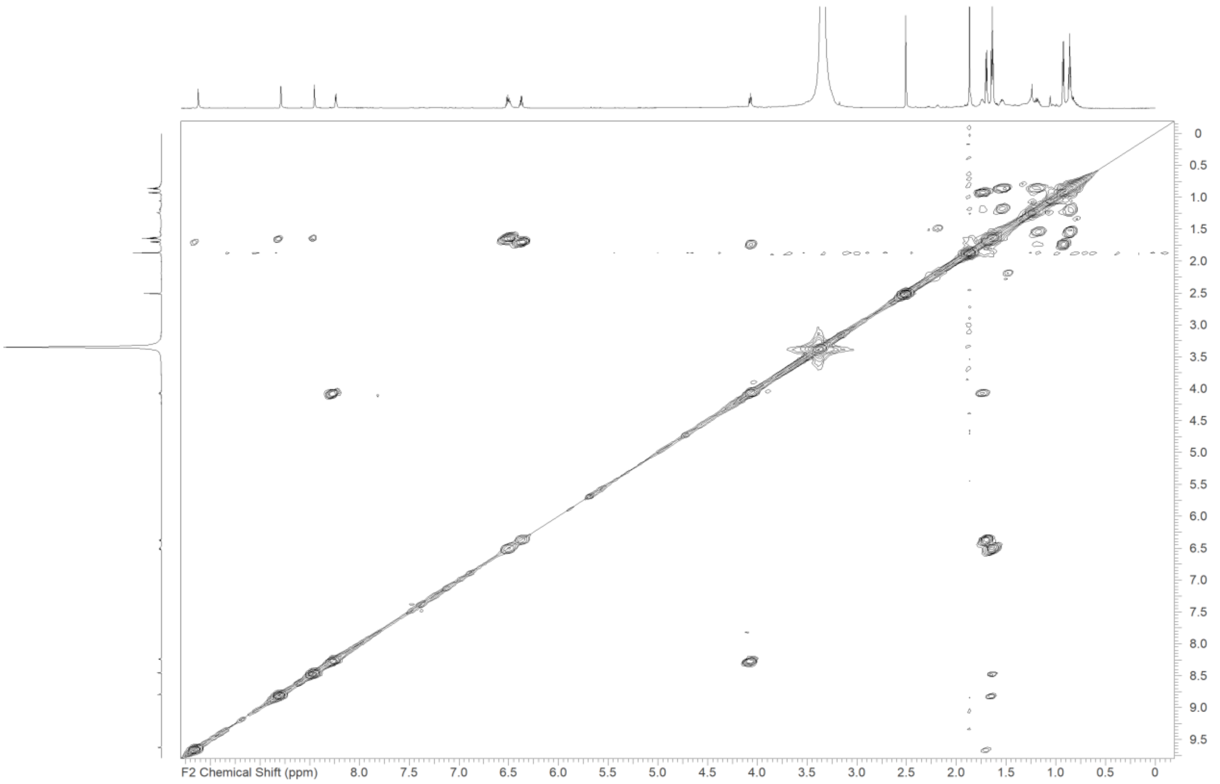

Figure S162. ^{1}H-^{1}H-Cosy spectrum of scabimycin C (DMSO-d_6, 700 MHz).

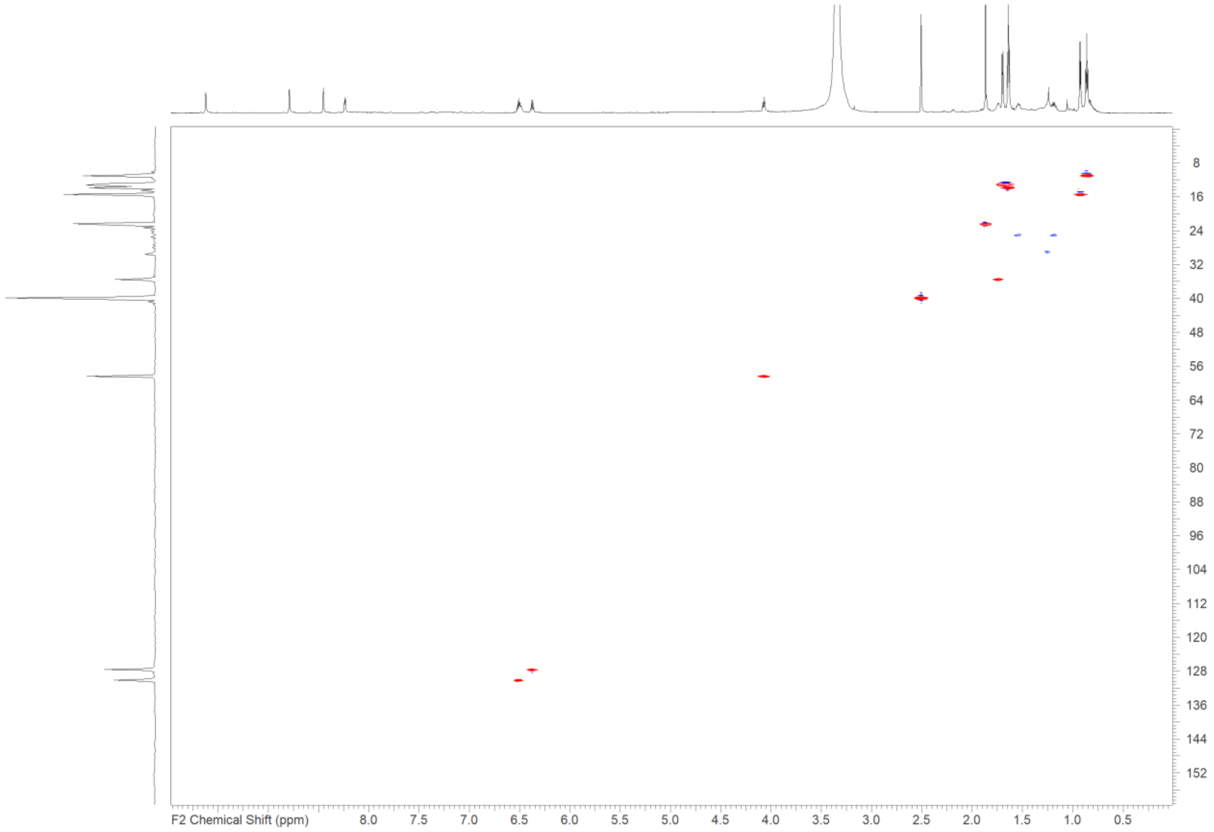

Figure S17. HSQC spectrum of scabimycin B (DMSO-d_6, 700 MHz).

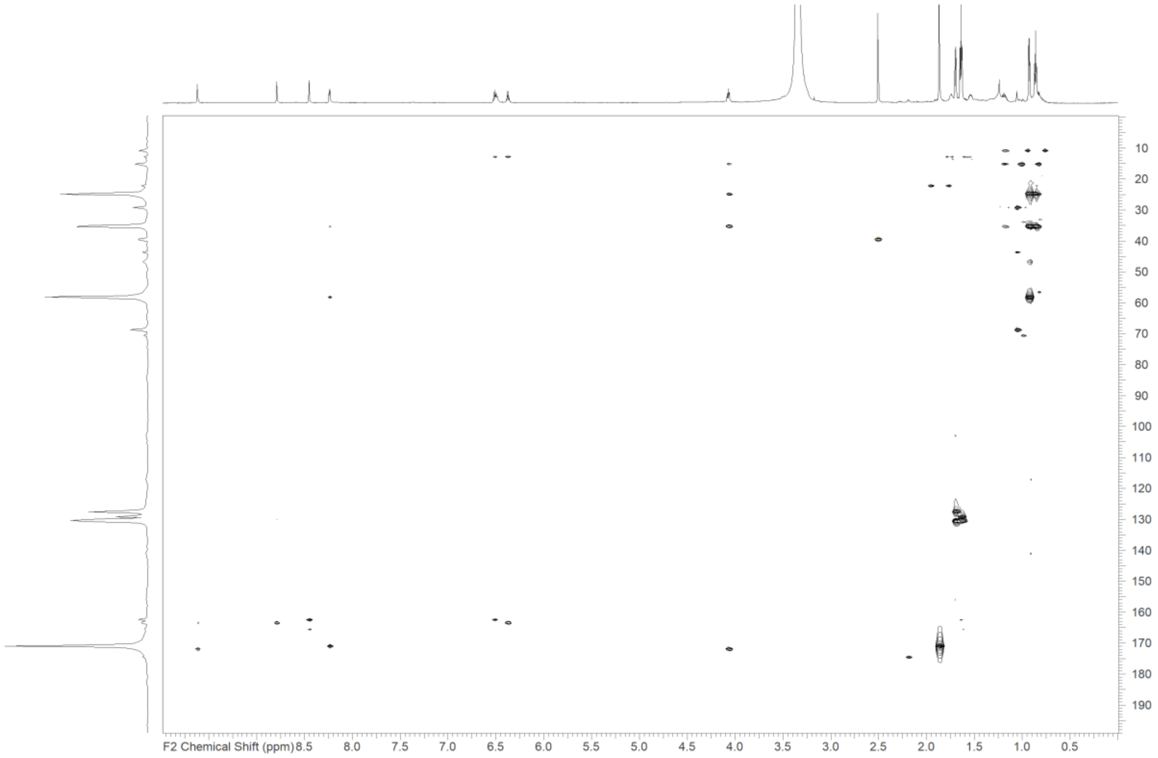

Figure S18. HMBC spectrum of scabimycin C (DMSO-d$_6$, 700 MHz).

Marfey's Method

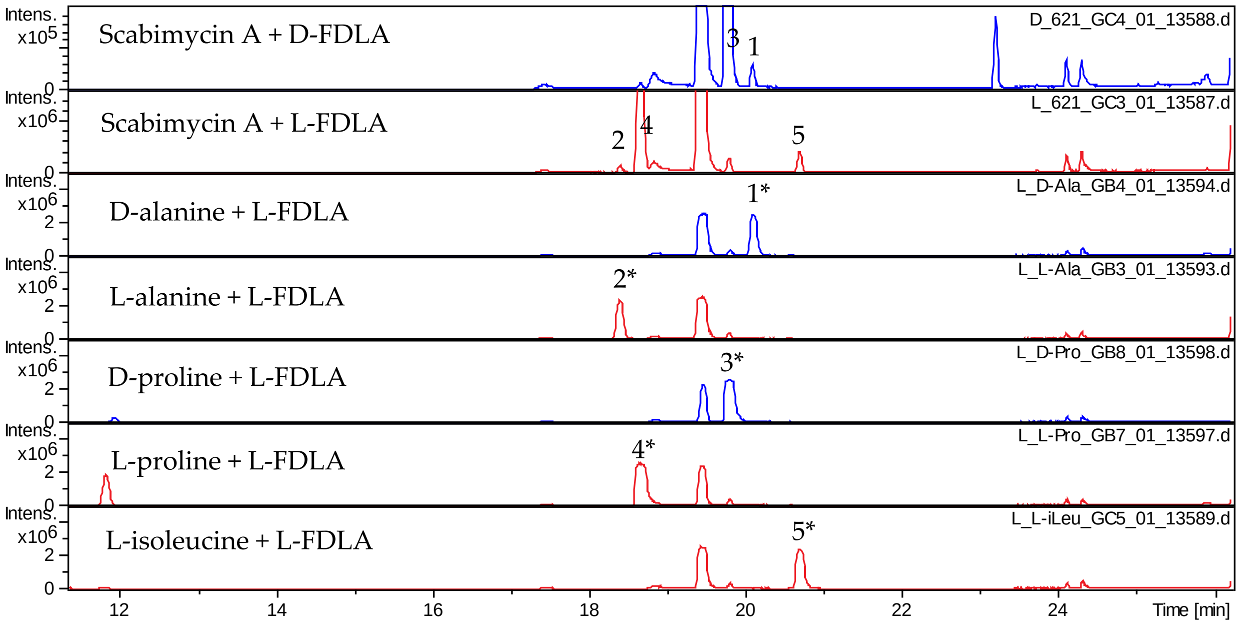

Figure S19. Extract of LC-HRMS chromatogram (retention time 17 to 28 min) of hydrolyzed scabimycin A derivatized with D-FDLA and L-FDLA showing single amino acids D/L-alanine (1/2), D/L-proline (3/4) and isoleucine (5) and leucine (1/3). To compare the amino acids present in our probe, pure D/L-amino acids derivatized with L-FDLA (D/L-alanine (1*/2*), D/L-proline (3*/4*) and L-isoleucine (5*)) are shown below. Comparison with the standard amino acids and the fact that D-FDLA derivatized amino acids elute later, lead us to the assumption that in all cases the amino acids

possess L-configuration. It needs to be mentioned that from this test, it is not possible to decide whether L-allo-isoleucine or L-isolelucine is present. The intensive peak at RT 19.5 coincides with the mass of the underivatized FLDA.

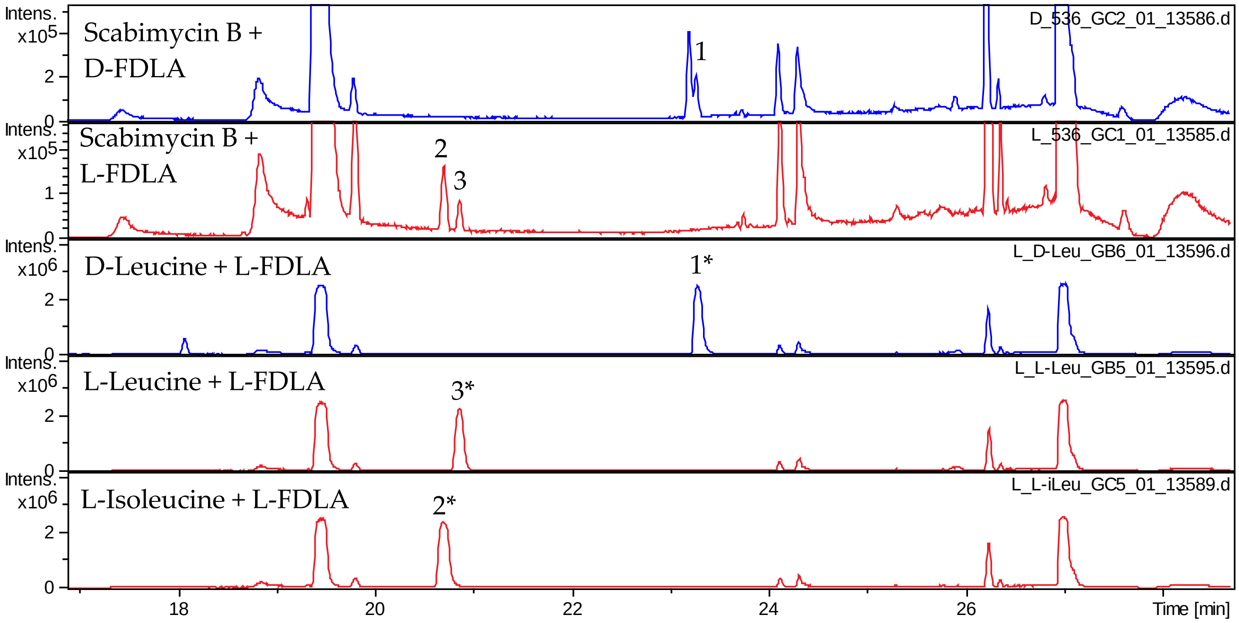

Figure S20. Extract of LC-HRMS chromatogram (retention time 17 to 28 min) of hydrolyzed scabimycin B derivatized with D-FDLA and L-FDLA showing single amino acids isoleucine (2) and leucine (1/3). To compare the amino acids present in our probe, pure D/L-amino acids derivatized with L-FDLA (D-Leucine (1*), L-Leucine (3*) and L-isoleucine (2*)) are shown below. Comparison with the standard amino acids and the fact that D-FDLA derivatized amino acids elute later, lead us to the assumption that both in both cases the amino acids have L-configuration. It needs to be mentioned that from this test, it is not possible to decide whether L-allo-isoleucine or L-isolelucine is present. The intensive peak at RT 19.5 coincides with the mass of the underivatized FLDA.

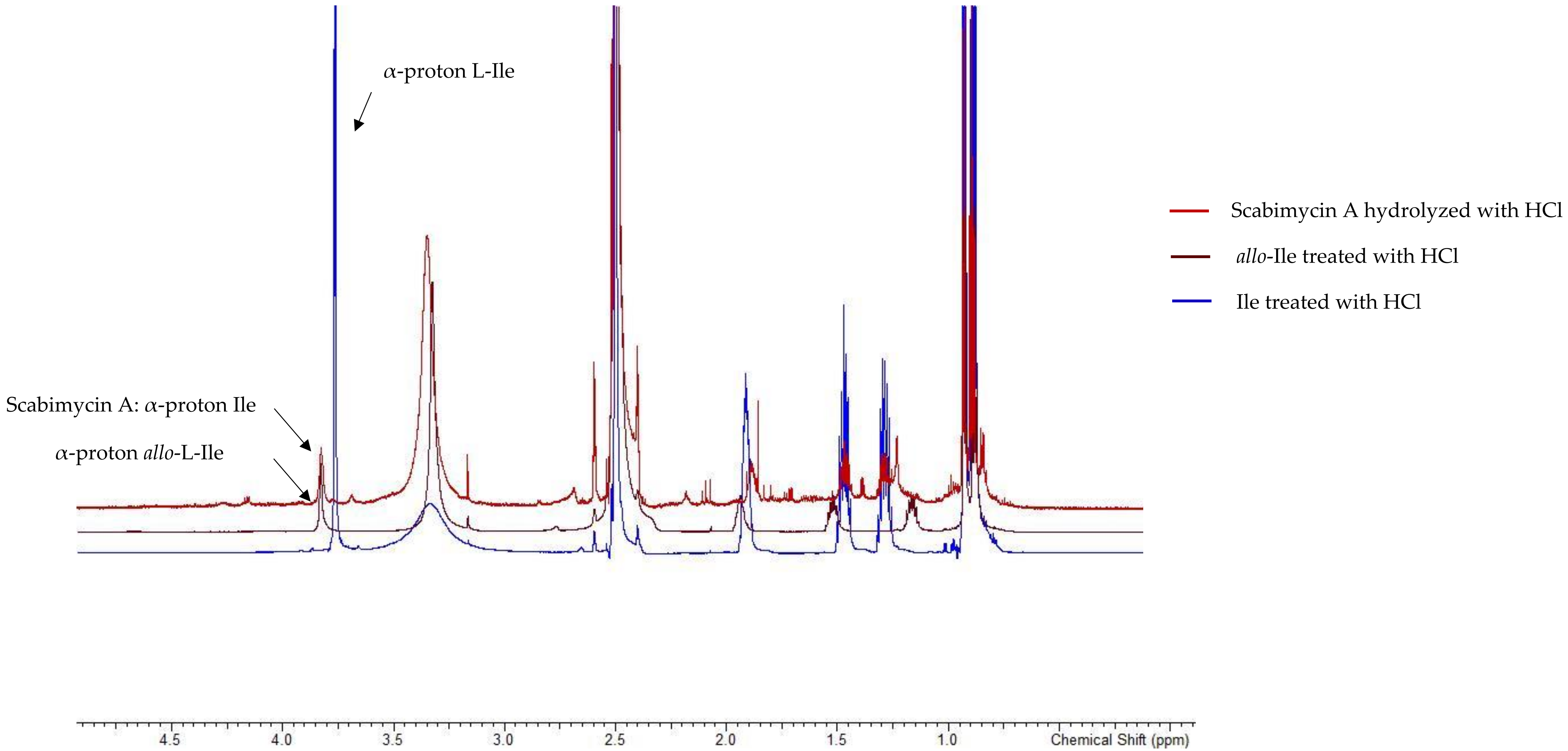

Figure S21. Determination of *allo*-isoleucine in scabimycin A. Scabimycin A, allo-L-Ile and L-Ile were treated with HCl at 110 °C. ^{1}H-NMR were acquired in DMSO-d_6 and compared. The chemical shifts of α-proton of scabimycin A and *allo*-L-Ile coincide while α-proton of L-Ile is shifted up-field.

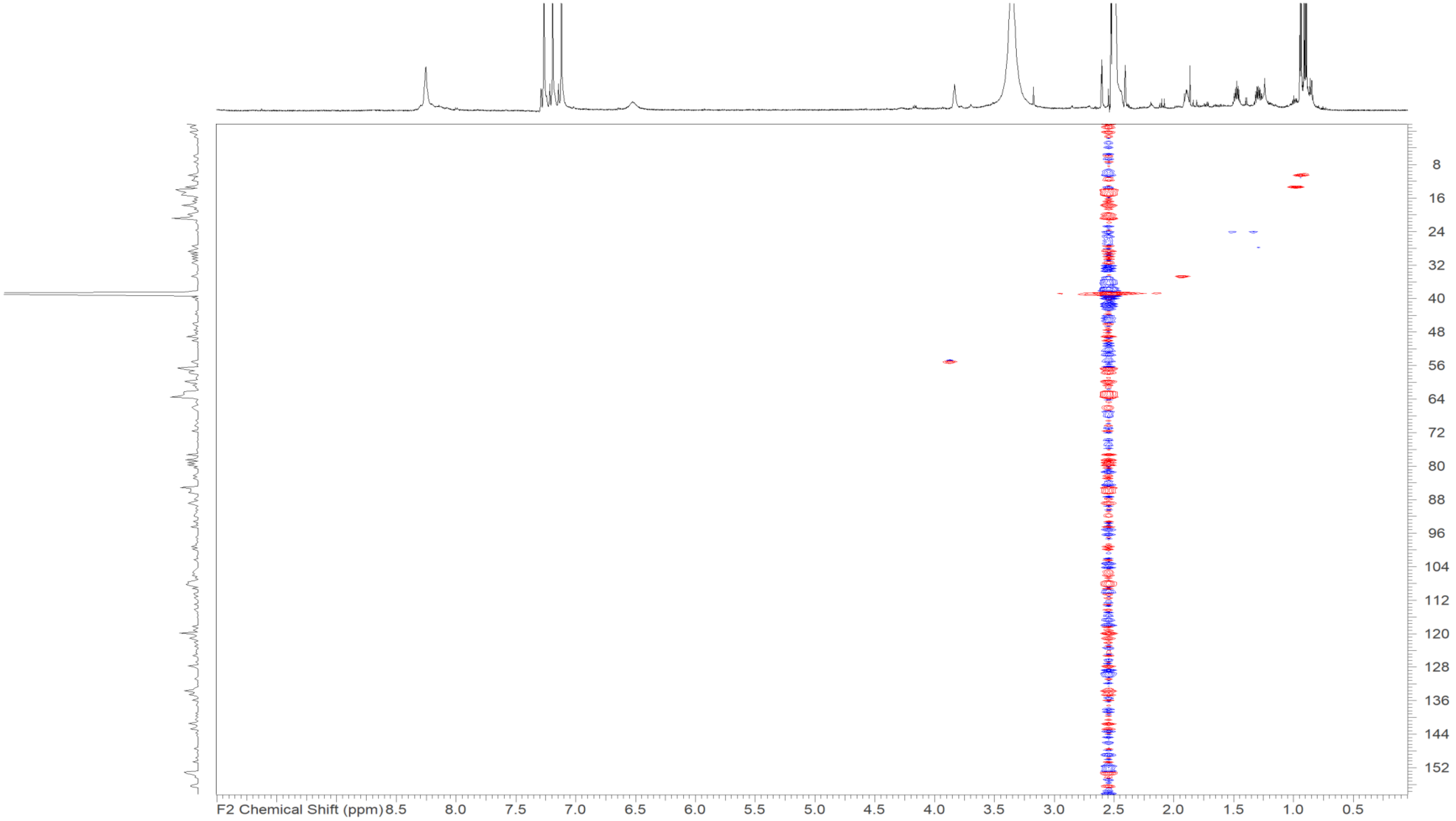

Figure S22. HSQC of scabimycin A after treatment with HCl to support the findings for allo-isoleucine. ^{13}C chemical shift for α-carbon was found to be around 55 ppm as expected for isoleucine.

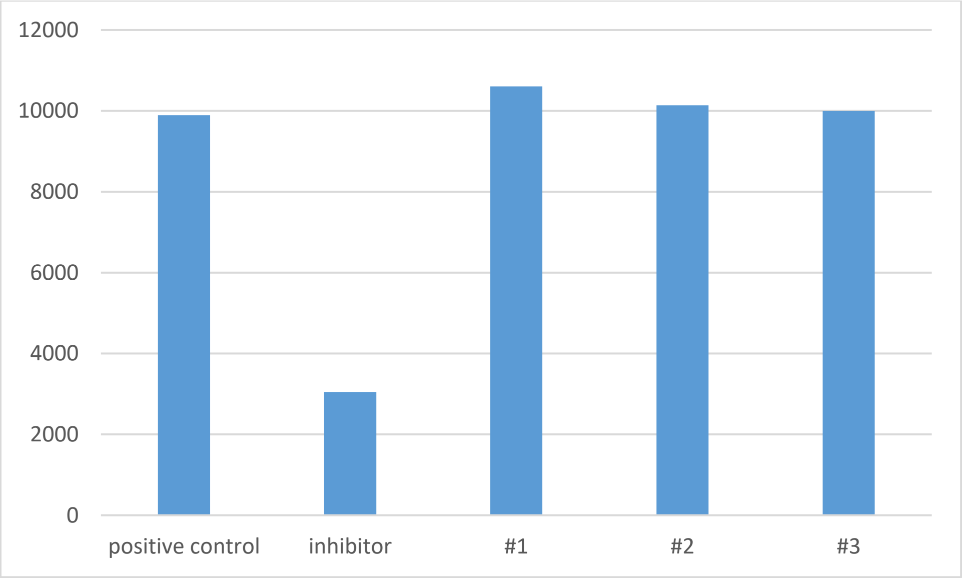

Figure S23. Inhibition of 3CL protease by scabimycin A. Positive control – activity of protease without inhibitor, Inhibitor – activity of protease after treatment with specific inhibitor, #1, #2 and #3 – activity of protease inhibited by scabimycin A dilutions #1, #2 and #3.
